# Modulating alternative splicing of *MECP2* is a potential therapeutic strategy for Rett syndrome

**DOI:** 10.1126/scitranslmed.adq4529

**Published:** 2026-03-04

**Authors:** Harini P. Tirumala, Li Wang, Yan Li, Sameer S. Bajikar, Ashley G. Anderson, Wei Wang, Alexander J. Trostle, Mahla Zahabiyon, Aleksandar Bajic, Jean J. Kim, Hu Chen, Zhandong Liu, Huda Y. Zoghbi

**Affiliations:** 1 Department of Human and Molecular Genetics, Baylor College of Medicine, Houston, TX 77030; 2 Jan and Dan Duncan Neurological Research Institute at Texas Children’s Hospital, Houston, TX 77030; 3 Department of Molecular and Cellular Biology, Baylor College of Medicine, Houston, Texas 77030; 4 Howard Hughes Medical Institute, Baylor College of Medicine, Houston, Texas 77030; 5 Department of Pediatrics, Baylor College of Medicine, Houston, Texas 77030; 6 Advanced Technology Cores, Baylor College of Medicine, Houston, Texas 77030; 7 Present address: Department of Biology, Stanford University, Stanford, CA 94305; 8 Present address: Departments of Cell Biology and Biomedical Engineering, University of Virginia, Charlottesville, VA 22903; 9 Present address: Department of Surgery, UT Southwestern Medical Center, Dallas, TX 75390

## Abstract

Rett syndrome (RTT) is a neurological disorder caused by loss-of-function mutations in methyl CpG binding protein 2 (*MECP2)*, a transcriptional regulator essential for maintenance of normal neuronal function. The current FDA-approved treatment for RTT, Trofinetide, mildly alleviates some symptoms. In contrast, re-introducing MeCP2 or increasing its amount through transgenesis in mouse RTT models improves most neurological phenotypes and enhances survival. Here, we devised a therapeutic strategy to moderately increase MeCP2 protein by modulating the alternative splicing of *MECP2* to switch the less efficiently translated *e2* to the more efficiently translated *e1* isoform. We deleted *Mecp2* exon 2 (unique to *e2*), leading to production of only *e1* mRNA, and show this upregulates MeCP2 by 50-60% in mice. Next, we investigated the consequences of isoform switching in two independent RTT induced pluripotent stem cell (iPSC)-derived neuron models harboring mutations that reduce both MeCP2 expression and function. Exon 2 deletion in MeCP2-G118E patient-derived neurons upregulated MeCP2, ameliorated morphological and electrophysiological changes and corrected the dysregulated transcriptome in these neurons. Isoform switching in MeCP2-T158M patient-derived neurons, modelling a severe RTT mutation, only modestly affected MeCP2 protein abundance and despite this, led to a partial transcriptomic rescue. Lastly, an exon 2-skipping Morpholino upregulated MeCP2-E1 *in vivo* in mice. These data set the stage for a potential therapeutic strategy using antisense oligonucleotides to promote isoform switching in patients with RTT who carry partially functioning alleles of *MECP2*.

## One Sentence Summary

Switching of *MECP2-e2* to the more efficiently translated *e1* isoform upregulates MeCP2 protein and rescues deficits in RTT iPSC-derived neurons.

## INTRODUCTION

Rett syndrome (RTT, OMIM: #312750) is a severe neurological disorder caused by loss-of-function mutations in the X-linked gene methyl CpG binding protein 2 (*MECP2*)(1). RTT mainly affects females with an incidence of ~1 in 10,000 live female births(2). The rare cases of RTT males carrying a null *MECP2* mutation typically die in infancy, but males with mutations that do not completely abolish MeCP2 function survive into childhood(3, 4). Female patients with RTT display apparently normal development up to 6-18 months of age followed by regression with loss of acquired language skills, purposeful hand movements, and cognitive and motor skills along with seizures and breathing abnormalities(5). Despite its severity, RTT does not cause neuronal death(6) and the brain circuitry remains intact, although dysfunctional (7, 8). Studies in *Mecp2*-null mice that recapitulate RTT features demonstrate that RTT pathology is reversible upon genetic reintroduction of normal MeCP2 in the brains of adult symptomatic mice, suggesting that RTT could be treated by restoring MeCP2 expression(9). Interestingly, in a mouse model carrying a highly prevalent RTT-causing mutation (T158M), overexpression of the mutant MeCP2 by ~4.5-fold improved RTT phenotypes including survival, motor coordination, motor learning, and respiratory abnormalities(10). This demonstrates that upregulation of mutant MeCP2 that retains a little function can improve disease phenotypes. This is important because ~65% of patients with RTT have partially functional MeCP2 that either has decreased DNA binding or is less abundant than normal (RTTBase)(11). Therefore, upregulating endogenous MeCP2 in these patients could provide therapeutic benefit.

Developing therapeutics that modulate MeCP2 abundance has been challenging because the brain is highly sensitive to MeCP2 dosage. Loss of MeCP2 function leads to RTT, whereas too much MeCP2 leads to another severe neurological disorder, *MECP2* Duplication Syndrome (MDS)(12). Moreover, *MECP2* undergoes X-inactivation in females which results in ~50% of the cells carrying mutant *MECP2* and the other 50% carrying wild-type *MECP2*(1). Therefore, in these females it is critical to ensure that therapeutic strategies to increase MeCP2 do not cause adverse effects due to *MECP2* overexpression in cells carrying wild-type *MECP2*. The two gene therapy approaches currently in clinical trials for RTT employ viral delivery of *MECP2* designed to be autoregulated by miRNAs to allow some control over MeCP2 abundance to minimize the risk of overcorrection(13, 14). However, current gene therapy vectors deliver only to 10% to 15% of cells, near the injection site, in non-human primate brain regions indicating limited biodistribution(15, 16). Given that MeCP2 is expressed throughout the brain(17) and that multiple brain regions are affected in RTT(18), it is necessary to explore additional therapeutic strategies that allow for broader distribution while ensuring the amount of MeCP2 remains within a tolerable range. Here, we explore this strategy by targeting the alternative splicing of *MECP2* and capitalizing on its effects on MeCP2 translation.

**Fig 1.**
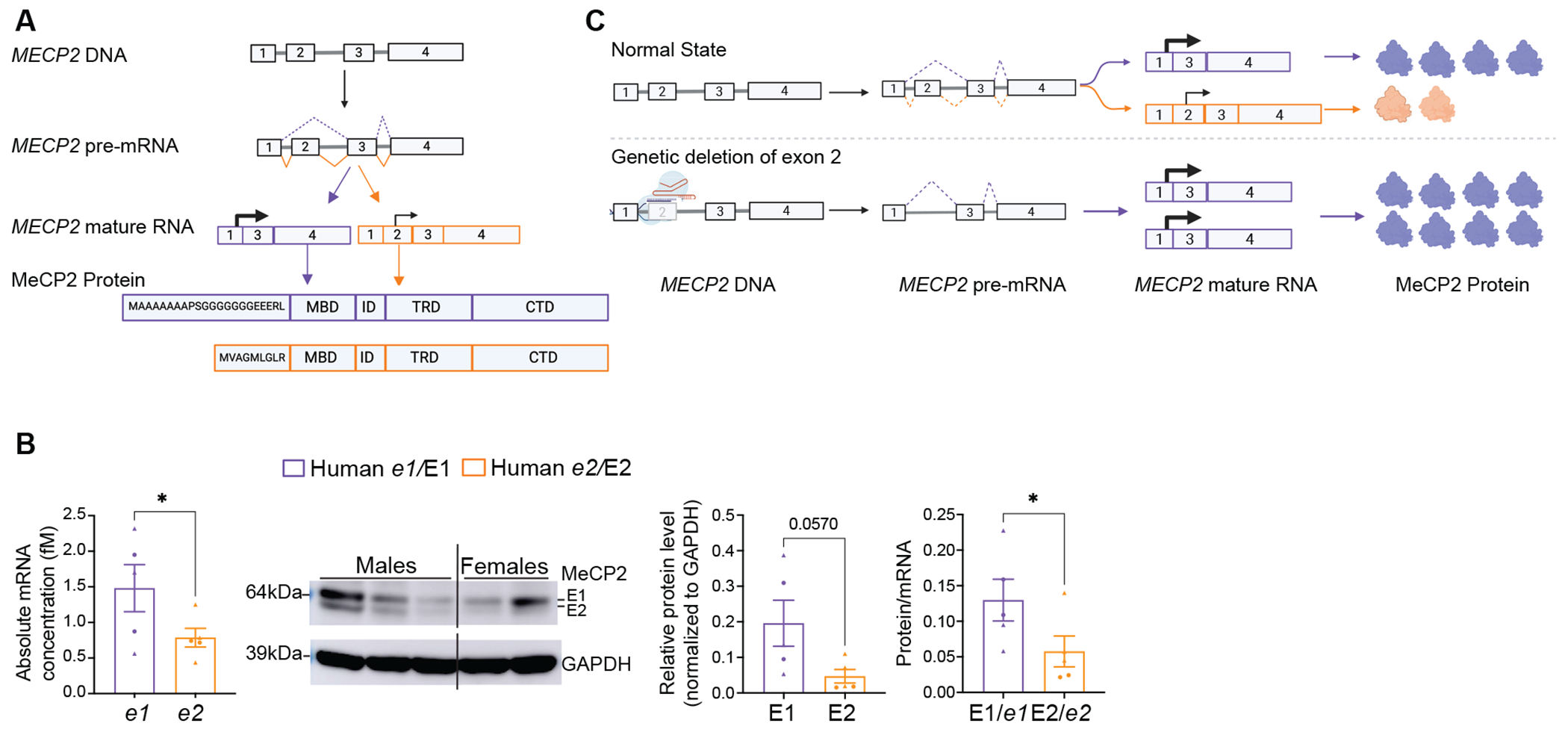
*MECP2-e1* and *e2* are alternatively spliced and differ in translational efficiency. (A) Schematic of *MECP2* exons alternatively spliced into *e1* and *e2* at pre-mRNA level. Purple denotes *e1* and orange denotes *e2*. Translational start sites of *e1* and *e2* are indicated by a black arrow on mature mRNA. E1 and E2 proteins have distinct N-termini but identical functional domains. (B) *e1* and *e2* absolute mRNA concentrations (left), Western blot showing E1 and E2 protein bands (center), E1 and E2 protein quantifications and protein/mRNA ratios (right) in post-mortem human prefrontal cortex tissues (n= 3 males (triangles) and 2 females (circles)), statistical analyses done using paired t-tests (*p<0.05). Data are presented as mean±sem. Error bars represent sem. Individual datapoints representing biological replicates. (C) Schematic representation of how isoform switching in *MECP2* by exon 2 deletion abolishes *e2* mRNA, upregulates *e1* mRNA, thereby upregulating E1 protein.

The four exons of *MECP2* are alternatively spliced into two mRNA isoforms: *MECP2-e1* which encompasses exons 1, 3, and 4; *MECP2-e2*, which includes all 4 exons of *MECP2*. Exon 2 is thus unique to the *e2* isoform(19, 20). The two isoforms are differentially expressed across different brain regions with *e1* being the predominant isoform(17). *e1* mRNA is slightly higher than *e2* across multiple tissues including the brain in humans and mice (17). MeCP2-E1 protein is translated from an ATG in exon 1 whereas MeCP2-E2 protein is translated from an ATG in exon 2, resulting in E1 and E2 having distinct N-termini but identical functional domains (Fig. 1A)(19, 20). Moreover, expressing either wild-type *e1* or *e2* in *Mecp2*-deficient mouse fibroblasts, Kriaucionis et al. observed that E2 is much lower than E1 and mutating the exon 1 ATG in the *e2* isoform dramatically increased E2 protein. This suggests that the less efficient translation of endogenous *e2* is due to interference from a short open reading frame (ORF) translated from the upstream exon 1 ATG(19).

E1 is slightly less stable and more loosely bound to double stranded DNA than E2. ChIP-seq experiments revealed enrichment of E1 at genes related to neuroactive ligand-receptor interactions whereas E2 was enriched at ribosomal genes. Proteomic analyses of mouse whole brain lysates revealed that E1 and E2 interact with unique partners involved in the same biological processes such as mRNA processing, mRNA splicing, chromatin regulation, CamKII-related processes and tubulin-based processes. E2 was also found to interact with proteins involved in nuclear processes and transcriptional regulation(21). Importantly, to date, no exon 2 mutations have been reported in patients with RTT, whereas mutations that exclusively disrupt the *e1* isoform cause RTT(22). Studies in mice demonstrate that loss of E1 alone is sufficient to recapitulate all the phenotypes observed in *Mecp2*-null mice(23, 24). Conversely, mice lacking E2 have decreased embryonic viability due to placental development problems. However, mice lacking E2 that make it to term survive normally and do not display RTT-like deficits(25). Together, these data show that the *e2* differs from *e1* by a single exon (exon 2), is less efficiently translated, is not associated with RTT mutations, and is dispensable for MeCP2 function in the postnatal brain. This led us to hypothesize that switching *e2* to the more efficiently translated *e1* at the mRNA level by skipping exon 2 can upregulate MeCP2 protein in patients with RTT to improve phenotypic outcomes.

To test our hypothesis, we genetically deleted exon 2 in wild-type mice to assess the impact of isoform switching on MeCP2 expression and neurological function. We then tested whether genetic deletion of exon 2 in two human RTT neuron models upregulates MeCP2 and improves their phenotypes. Finally, to assess the therapeutic potential of the isoform switching, we tested an exon 2-skipping morpholino in mice and assessed its effect on MeCP2 protein.

## RESULTS

### *MECP2-e2* mRNA is abundant in the human prefrontal cortex and is less efficiently translated than *e1*

*MECP2-e1* and *e2* are alternatively spliced and differ by a single exon (exon 2) (Fig 1A). To determine whether there is sufficient *e2* at the mRNA level to switch into *e1* in the postnatal human brain, we measured the absolute concentrations of *MECP2 e1* and *e2* mRNA in postmortem human prefrontal cortex (PFC) (Fig. 1B). We found that *e2* abundance is about half that of *e1*, indicating sufficient *e2* for isoform switching to *e1*. At the protein level, we found E2 is lower than E1, and the protein:mRNA ratio of E1 is significantly higher than E2 (Fig. 1B, p=0.0332), confirming that *e2* is less efficiently translated than *e1*.

### Isoform switching moderately upregulates MeCP2 protein and leads to mild MeCP2-overexpression-like phenotypes in mice

**Fig. 2.**
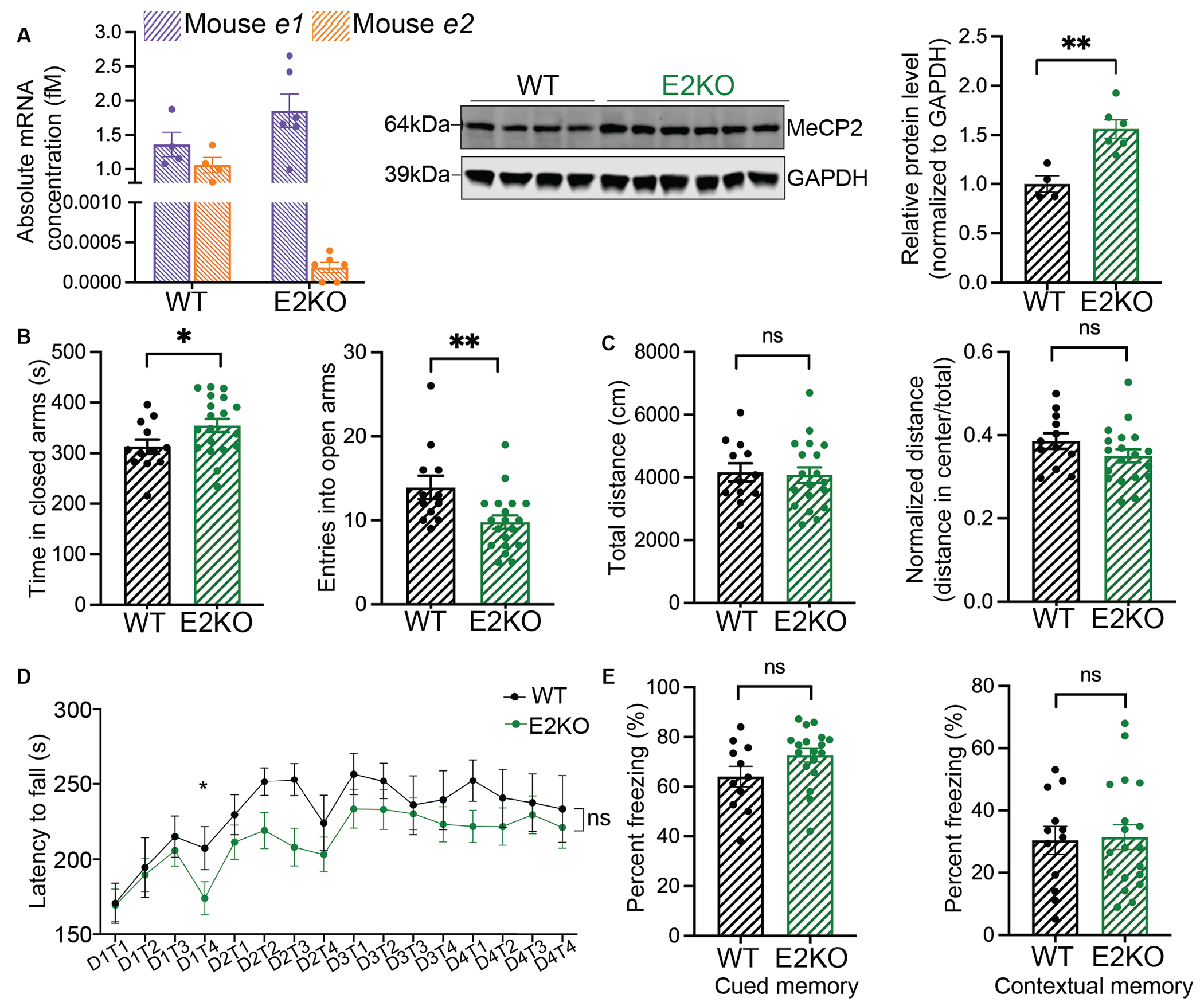
Isoform switching moderately upregulates MeCP2 protein leading to mild MeCP2-overexpression-like phenotypes in male mice. (A) *e1* and *e2* absolute mRNA concentrations (left), Western blot showing MeCP2 protein bands (center) and quantification (right) in cortices of wild-type–black (n=4) and E2KO–green (n=6) mice (B) Elevated plus maze (EPM) analysis: time in closed arms (left) and number of entries into open arms (right) (C) Open field assay (OFA): total distance travelled (left) and normalized distance (distance in center/total distance) (right) (D) Rotarod assay: latency to fall (in seconds) across 4 test days, 4 trials per day (E) Percent freezing in the conditioned fear paradigm in response to cue (left) and context (right). Behavioral assays were performed on 12 wild-type and 19 E2KO male mice. Statistical analyses: panels A,B,C, E: unpaired t-test and panel D: two-way ANOVA with multiple comparisons (ns: p>0.05, * p<0.05, ** p<0.001). Data are presented as mean±sem. Error bars represent sem. Individual datapoints represent biological replicates.

To test whether isoform switching of *e2* to *e1* (Fig. 1C) would upregulate MeCP2 protein and to characterize its effect on neurological behavior, we generated a mouse line carrying a deletion of *Mecp2* exon 2 (E2KO mouse). The exon 2 knockout mouse previously generated by Itoh et *al.*, was assessed for embryonic viability but not for MeCP2 protein abundance or in-depth behavioral characterization(25). Therefore, we measured *Mecp2-e1* and *e2* mRNA and their encoded proteins in the cortex and other brain regions in adult male E2KO mice. At the mRNA level, we observed complete loss of *e2* and upregulation of *e1* in the cortices of E2KO mice compared to wild-type mice (Fig. 2A). Consequently, MeCP2 protein was ~50-60% higher in the cortices of E2KO mice than wild-type mice (Fig. 2A). The loss of *e2* mRNA and upregulation of MeCP2 protein were consistent across other brain regions (Fig. S1A–C). The increase in MeCP2 was driven specifically by an increase in the E1 isoform, with complete loss of E2 in the E2KO mice. These results show that switching *e2* to *e1* leads to increased amounts of MeCP2 protein.

Next, we performed behavioral assays to assess the functional consequences of increasing MeCP2 by ~50-60%. A 100% increase in MeCP2 leads to abnormal behavioral phenotypes in mice modelling MDS (*MECP2*-Tg1 mouse)(26). These mice display heightened anxiety-like behavior, enhanced motor learning and enhanced memory phenotypes that are reciprocal in direction compared to RTT mouse models (27). We tested 10-week-old E2KO male mice and wild-type littermates on the elevated plus maze and observed that E2KO male mice displayed increased anxiety-like phenotypes (Fig. 2B). However, they did not display any deficits in locomotor activity (Fig. 2C), or motor learning and memory (Fig. 2D). E2KO mice also did not show any changes in amygdala-dependent (cued) or hippocampus-dependent (contextual) learning in the fear conditioning assay (Fig. 2E). Therefore, while a 100% increase in MeCP2 causes multiple phenotypes (26, 27), a 50-60% increase in every cell only led to mild deficits manifesting as increased anxiety-like behavior. Given that most patients with RTT are female, we rationalized that it is important to assess the impact of a moderate upregulation of wild-type MeCP2 in ~50% of the cells in female mice. So, we performed behavioral analyses on 15–16-week-old *Mecp2^E2KO/+^* female mice and found that these mice do not display any deficits in anxiety (Fig. S1D) or locomotion (Fig. S1E). Taken together, moderately upregulating wild-type MeCP2 by switching *e2* mRNA to *e1* in mice is well-tolerated, particularly in the mosaic female mice.

### Isoform switching in RTT neurons upregulates MeCP2

**Fig. 3.**
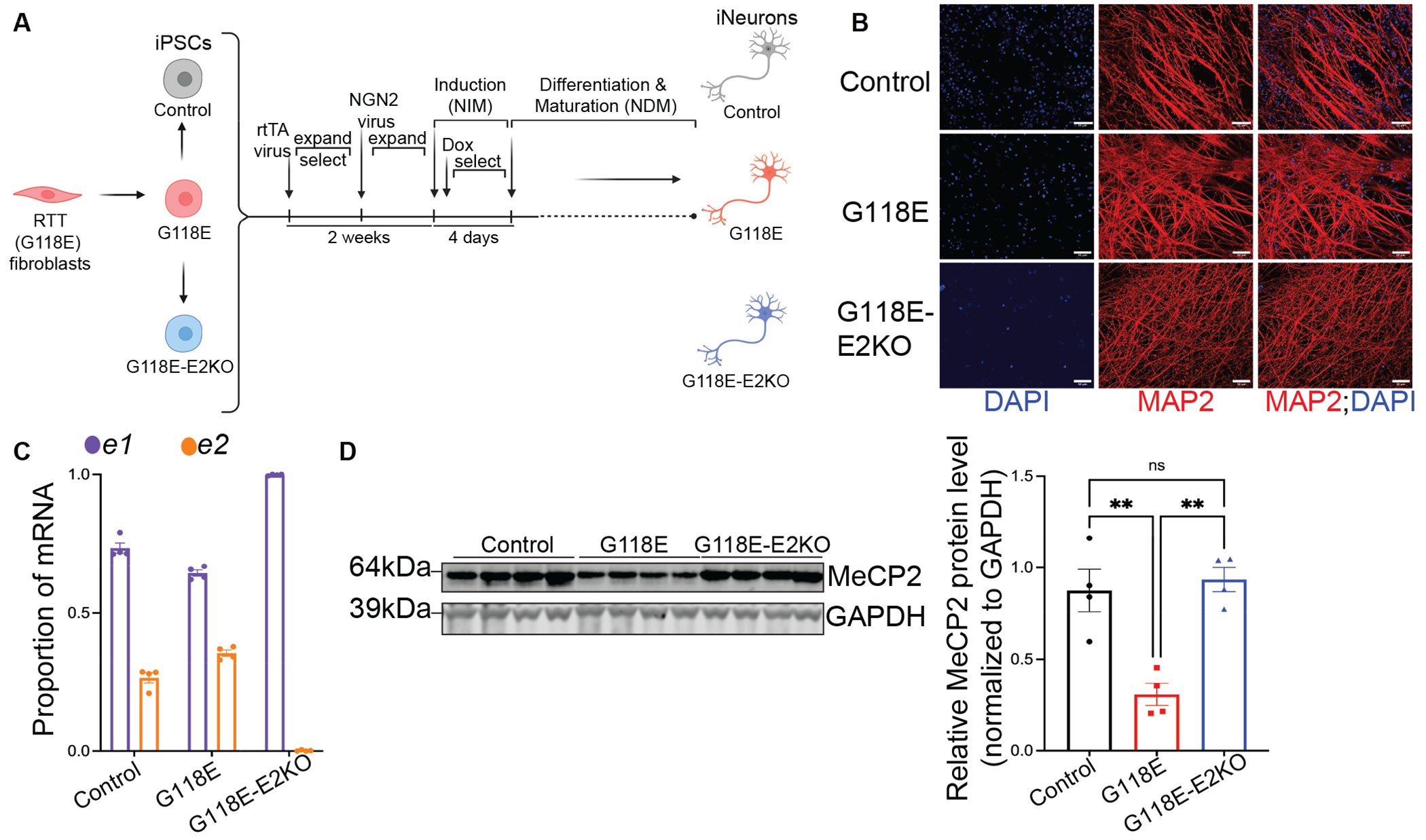
Isoform switching in G118E NGN2-iNeurons upregulates MeCP2 protein to control range. (A) Schematic of G118E, control and G118E-E2KO NGN2-iNeurons derivation from G118E patient fibroblasts. G118E fibroblasts were reprogrammed into G118E iPSCs (in red), which were then edited by CRISPR/Cas9 editing to correct the G118E mutation to WT to generate isogenic control iPSCs (in grey). Exon 2 was deleted by CRISPR/Cas9 editing in G118E iPSCs to generate G118E-E2KO iPSCs (in blue). These three sets of iPSCs were sequentially infected with lentivirus containing doxycycline-inducible rtTA and NGN2 constructs and exposed to doxycycline to overexpress NGN2 and induce neuronal differentiation to generate NGN2-iNs. Figure created with Biorender.com. (B) Representative immunofluorescence images of NGN2-iNeurons (Control, G118E and G118E-E2KO) at 8 weeks of differentiation stained for the neuronal maturation marker MAP2 (red) and DAPI nuclear stain (in blue) (Scale bar: 50μm) (C) *e1* (purple) and *e2* (orange) mRNA proportions measured by qRT-PCR in control, G118E and G118E-E2KO NGN2-iNeurons (D) Left – Western blot showing MeCP2 and GAPDH (internal control) proteins in control, G118E and G118E-E2KO NGN2-iNs (N=4 technical replicates per genotype), right - MeCP2 quantification relative to GAPDH. Statistical analysis in panel D was performed by two-way ANOVA with multiple comparisons (ns: p>0.05, * p<0.05, ** p<0.01). Data are presented as mean±sem. Error bars represent sem. Individual datapoints represent replicate wells.

To test if exon 2 deletion would upregulate MeCP2 in a human RTT model, we first deleted exon 2 in iPSCs derived from a male patient carrying the G118E mutation. This mutation led to global developmental delay, hypotonia, motor planning difficulties, seizures(4), and premature death at 6 years of age. It reduces MeCP2 in mice and human iPSC-derived neurons, disrupts DNA-binding, and causes RTT-like features in mice(4). The G118E mutation is ideal to test our hypothesis because it affects both MeCP2 abundance and DNA binding. Therefore, we deleted exon 2 in *MECP2^G118E/y^* patient-derived iPSCs (Fig. S2A–C) and conducted studies on six lines of iPSCs with two clones for each of the three genotypes: *MECP2^G118E/y^* (G118E), *MECP2^G118E-E2KO/y^* (G118E-E2KO) and isogenic control (control) expressing normal *MECP2*. First, we confirmed these iPSCs show normal karyotype (Data file S1) and display self-renewal and pluripotent properties (Fig. S2D). The primary clinical phenotypes in RTT are neurological. Hence, we differentiated these six iPSC lines into neurons by two independent, well-established neuronal differentiation methods to ensure reproducibility of exon 2 deletion effects on neuronal phenotypes: 1) Neurogenin 2 overexpression(28) (NGN2-iNs) (Fig. 3A), and 2) dual-SMAD inhibition (29) (NPC-iNs) (Fig. S3A). Staining for neuronal maturity markers after 8 weeks of differentiation, we found that one whole set (one clone of each genotype) of the NGN2-iNs highly expressed the neuronal cytoskeletal protein MAP2 (Fig. 3B) but the second set of NGN2-iNs (one clone per genotype) showed very sparse expression of this marker. Therefore, we assessed only one set of these NGN2-iNs for electrophysiology and neuronal morphology analyses. We also stained NPC-iNs after 12 weeks of differentiation and showed that all six lines highly express MAP2 and Beta III tubulin (Fig. S3B).

The G118E mutation does not affect *MECP2* mRNA but destabilizes MeCP2 protein(4). Measuring mRNA and protein of MeCP2 in 8-week-old NGN2-iNs, we therefore did not see a difference in the proportions of *e1* and *e2* mRNA in the G118E neurons relative to control (Fig. 3C); however, there was a significant decrease of MeCP2 protein in the G118E neurons relative to control (Fig. 3D, p=0.0027). In the G118E-E2KO neurons, *e2* mRNA was completely absent and *e1* mRNA was upregulated (Fig. 3C). Correspondingly, MeCP2 protein was significantly increased in G118E-E2KO neurons (p=0.0014) and restored to an amount comparable to that of control neurons (Fig. 3D). In the G118E-E2KO NPC-iNs, *e2* mRNA was completely absent, *e1* mRNA was upregulated (Fig. S3C) and MeCP2 protein was also significantly increased relative to the G118E NPC-iNs (Fig. S3D, p=0.0328).

To test our isoform switching strategy in a commonly occurring severe RTT mutation, T158M(30), that reduces MeCP2 by ~65-70% and severely impairs its DNA-binding ability(10), we obtained MeCP2-T158M (T158M-MU) and isogenic control (T158M-WT) iPSCs derived from the same female patient. We genetically deleted exon 2 in the T158M-MU iPSCs as above to generate T158M-E2KO iPSCs (Fig. S4A) and differentiated the T158M-WT, T158M-MU and T158M-E2KO iPSCs (one clone per genotype) into neurons (by NGN2-overexpression, see Methods). Measuring mRNA and protein of MeCP2, we found that *e2* mRNA was abolished in the T158M-E2KO neurons with an upregulation of *e1* mRNA compared to the T158M-MU neurons (Fig. S4B). The T158M-MU neurons showed a ~70% reduction in MeCP2 compared to T158M-WT, and there was minimal upregulation of the mutant protein in the T158M-E2KO neurons compared to the T158M-MU neurons (Fig. S4C). Given that the T158M mutation severely impairs DNA-binding and stability of MeCP2, this small upregulation of mutant protein in the T158M-MU neurons by E2KO is anticipated.

### Isoform switching corrects transcriptomic dysregulation in RTT neurons

**Fig. 4.**
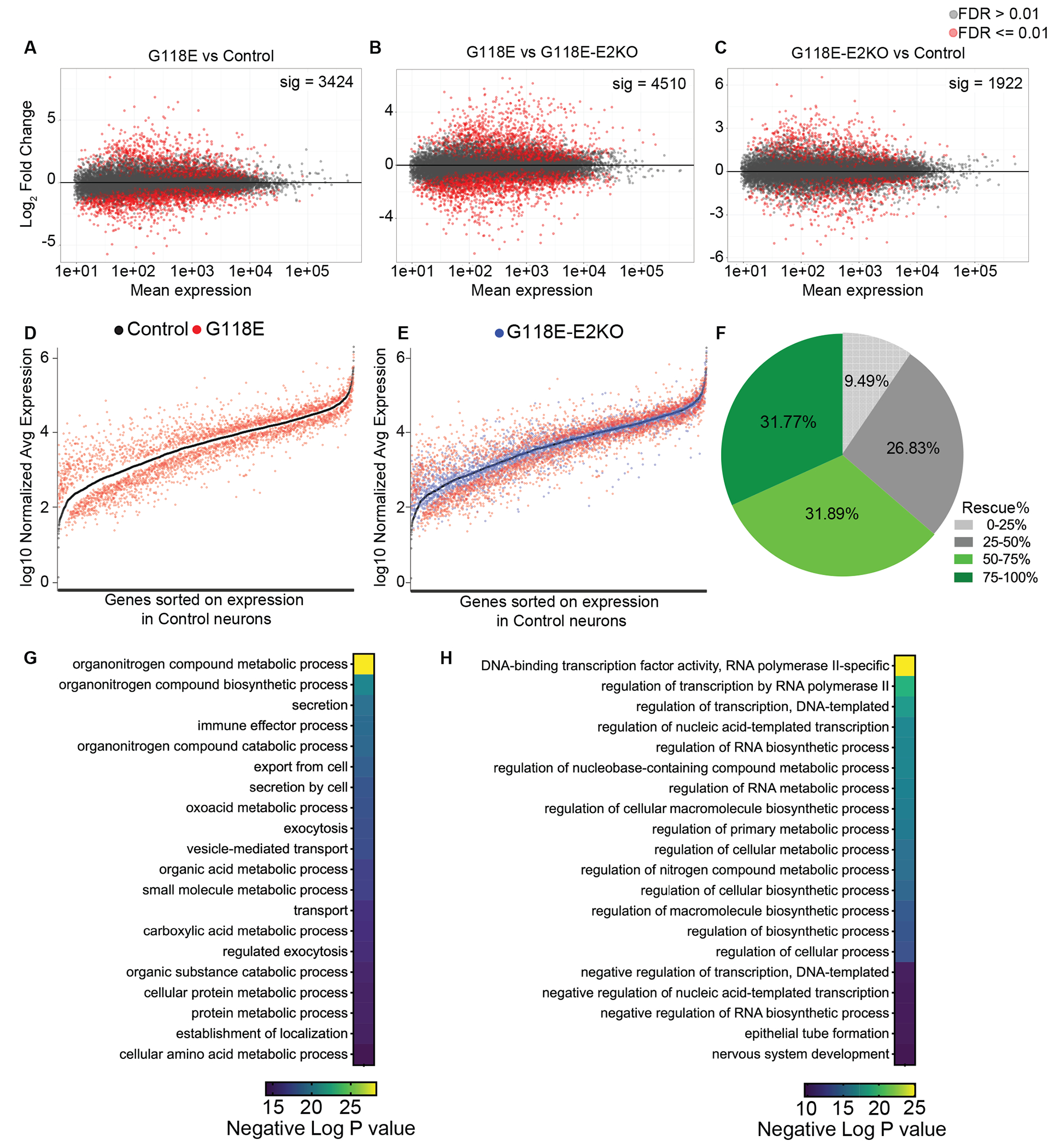
Isoform switching corrects transcriptomic dysregulation in 8-week-old G118E NGN2-iNeurons. (A-C) Volcano plots of DEGs between G118E and control (A) , G118E and G118E-E2KO (B) G118E-E2KO and control (C) NGN2-iNs with mean expression on X-axis and Log_2_ Fold change on Y-axis, (D-E) Dot plots of G118E disease gene expression (3,424 genes) with Log_10_Normalized average expression on Y-axis and individual genes arranged in ascending order based on expression in control iNeurons on the X-axis. Each X position corresponds to one gene. Expression in control: black, G118E: red and G118E-E2KO: blue (F) Pie-chart indicating magnitude of rescue in the corrected genes divided into four quartiles based on rescue percentage with each slice representing a range of rescue (light grey - 0-25%, dark grey - 25-50%, light green - 50-75% and dark green - 75-100%) and percent of genes indicated within each slice (G-H) Gene Ontology analysis of genes upregulated in G118E (G) and downregulated in G118E (H) and corrected in G118E-E2KO iNeurons showing significantly enriched biological processes on Y-axis and the color of the box indicating −log_10_(p_adj_) value with the scale shown below each panel.

Studies in mouse tissues(31, 32) and human iPSC-derived neuron models(33, 34) of MeCP2 disorders (RTT and MDS) have shown that the transcriptome is acutely sensitive to MeCP2 dosage. Moreover, transcriptomic changes occur earliest in the phenotypic cascade preceding circuit-level and behavioral dysfunction in RTT models(35–37) and are the first to rescue when reversing MeCP2-induced pathology due to duplication(32), highlighting their value as readouts in evaluating therapeutic efficacy. Accordingly, we hypothesized that the impact of the G118E mutation on the transcriptome in neurons could be rescued upon increasing mutant MeCP2 by exon 2 deletion, even though it has reduced ability to bind DNA(4). We performed bulk RNA-sequencing on the six clones of NGN2-iNs (two clones per each genotype) at two timepoints – 4 weeks and 8 weeks after differentiation. To examine if G118E-E2KO neurons were more similar to control neurons than the G118E neurons at the global transcriptome level, we calculated Pearson correlation coefficients between samples of all three genotypes and generated similarity matrices. The correlation coefficient of G118E-E2KO to control samples was 0.924 at 4 weeks (Fig. S5A) and 0.904 at 8 weeks (Fig. S5B) whereas the correlation coefficient of G118E-E2KO to G118E samples was 0.880 at 4 weeks (Fig. S5A) and 0.862 at 8 weeks (Fig. S5B). This suggests that the transcriptomic signature of G118E-E2KO neurons shares more similarity with the control than with the G118E neurons at both timepoints. Based on differences in neuronal health across the NGN2-iN clones, the two sets of clones (with one clone per genotype in each set) were designated as “lower health” and “higher health” (see Supplementary Methods). Principal component analysis (PCA) showed that neuronal health contributed to majority of the variance with lower and higher health clones of the same genotype separating out on the PCA (Fig. S6A). Therefore, we regressed out the effect of neuronal health on gene expression changes (see Supplementary Methods). We observed that the G118E neurons showed a marked dysregulation of gene expression across thousands of genes (FDR<=0.01) at both 4 weeks (2900 differentially expressed genes (DEGs)) and 8 weeks (3424 DEGs) (Fig. 4A) compared to control neurons. We also observed thousands of DEGs between G118E and G118E-E2KO neurons at 4 weeks (5239 DEGs) and 8 weeks (4510 DEGs) (Fig. 4B). The G118E-E2KO neurons had 2299 DEGs at 4 weeks and 1922 DEGs at 8 weeks (Fig. 4C) compared to the control neurons. To dissect the extent of transcriptomic rescue, we defined disease gene signature as the significant DEGs (FDR<=0.01) between the G118E and control neurons and asked how exon 2 deletion modulates this signature. Plotting the normalized log_10_ average expression of disease genes in control, G118E and G118E-E2KO neurons, we saw that the global dysregulation seen in G118E was largely corrected towards control expression in the G118E-E2KO neurons at both 4 weeks (Fig. S6B) and 8 weeks (Fig. 4D–E). Further quantification (see Supplementary Methods) demonstrated that ~60% of dysregulated genes were at least partially rescued in G118E-E2KO neurons at 4 weeks, which increased to ~65% at 8 weeks. Of these, 31.77% were normalized close to control neurons expression (>75% rescue) in the G118E-E2KO neurons at 8 weeks (Fig. 4F). Functional enrichment analysis on the rescued genes showed that in 4-week-old neurons, exon 2 deletion rescued genes involved in neuronal development, dendritic cell differentiation, cellular transport, and metabolic processes (Fig. S6C). In 8-week-old neurons, genes upregulated in G118E and rescued in the G118E-E2KO neurons are involved in key cellular and protein metabolic and transport processes (Fig. 4G), and those downregulated in G118E and rescued in the G118E-E2KO neurons are associated with transcription regulation and nervous system development (Fig. 4H). These data suggest that the disease genes rescued in the G118E-E2KO neurons are involved in several core neurological and cellular processes known to be regulated by MeCP2(38). We also performed bulk RNA-sequencing studies on the six lines of G118E, G118E-E2KO and isogenic control NPC-iNs differentiated for 14 weeks and performed similar analyses as described above for the NGN2-iNs. We found 8037 DEGs between control and G118E, 3457 DEGs between G118E and G118E-E2KO and 7735 DEGs between G118E-E2KO and control (Fig. S7A–C). We found that E2KO at least partially rescued ~62.5% of the 8037 G118E ‘disease genes’ (Fig. S7D–E). Examining the magnitude of rescue, we found that the majority of the genes whose expression improved (68.5%) were rescued by ≤50% indicating a more moderate magnitude of rescue in the NPC- iNs compared to the NGN2-iNs (Fig. S7F). Functional enrichment analysis revealed that the disease genes in NPC-iNs rescued in the G118E-E2KO neurons are involved in nervous system development, neurogenesis, and cell cycle processes (Fig. S7G).

To examine the consequences of exon 2 deletion in the context of the severe T158M mutation, we performed bulk RNA-sequencing on the T158M-WT, T158M-MU and T158M-E2KO iNeurons. Analyses were performed as detailed as for the G118E neurons dataset. PCA showed that majority of the variance among samples is explained by the genotype, with the T158M-E2KO neurons clustering between the T158M-WT and T158M-MU on the X-axis (Fig. S8A). We found that of the 1722 genes that were dysregulated in the T158M-MU neurons, 66.4% (1144 genes) were at least partially rescued in expression in the T158M-E2KO neurons (Fig. S8B). Examining the magnitude of rescue, 45.9% of the genes were rescued by more than 50% magnitude (Fig. S8C) implying that these genes were closer in expression to the T158M-WT than the T158M-MU neurons.

In addition to the T158M mutation, we took advantage of a recently published RNA-sequencing dataset in 3-week-old iNeurons carrying the R133C mutation in *MECP2*(39), one of the most frequent RTT mutations(30), which causes ~55% reduction in MeCP2 (40). Comparing DEGs in the R133C neurons (3200 DEGs) with the G118E DEGs (2900 DEGs) from our 4-week dataset we found 412 genes overlapped between the two datasets (Fig. S8D). In the G118E-E2KO neurons, we found that exon 2 deletion rescued the expression of 204 of these 412 genes (49.5%) (Fig. S8D). This suggests that boosting MeCP2 protein by exon 2 deletion rescues expression of at least a subset of dysregulated genes across multiple *MECP2* mutations.

Taken together, our transcriptomic analyses reveal that both G118E and T158M mutations of MeCP2 cause marked transcriptomic dysregulation in human iPSC-derived neurons and that this disease signature is partially normalized by isoform switching.

### Isoform switching ameliorates electrophysiological and morphological abnormalities in RTT neurons

**Fig. 5.**
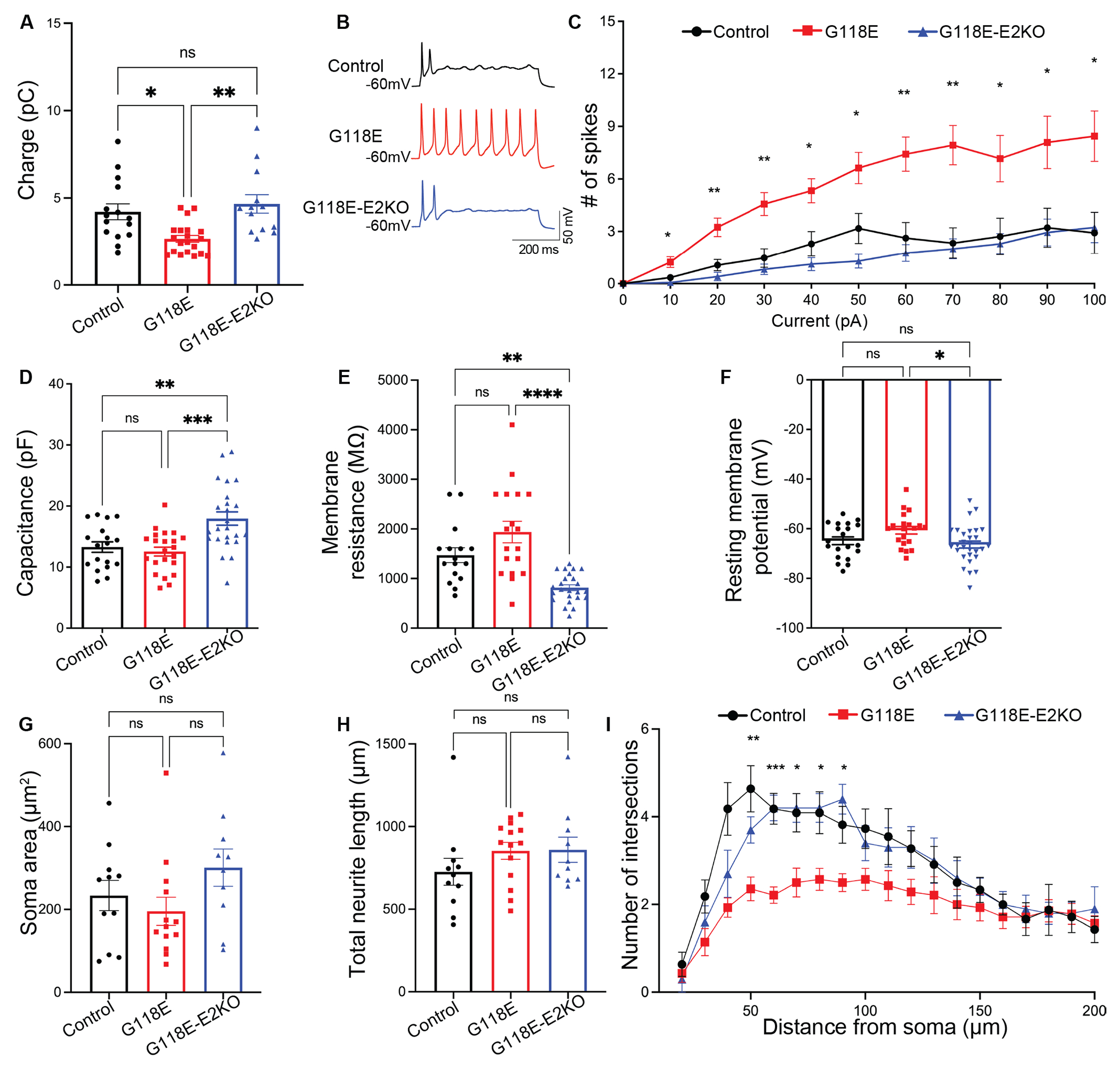
Isoform switching corrects electrophysiological and morphological deficits in G118E NGN2-iNeurons. (A) Box plots showing spontaneous synaptic charge transfer (charge, a measure of spontaneous synaptic current) of control, G118E and G118E-E2KO NGN2-iNeurons recorded under a voltage clamp. Dots show individual neurons (B) Representative traces of current injection-dependent firing (C) Firing in response to injection of increasing currents (D to F) Box plots showing membrane capacitance (D) Membrane resistance and (E) Resting membrane potential (F) of control, G118E and G118E-E2KO neurons. (G) Soma area (H) total neurite length and (I) quantification of dendritic arborization with number of intersections (Y-axis) plotted at increasing distances from the soma (X-axis) of control, G118E and G118E-E2KO neurons. Electrophysiology: n=16-29 neurons recorded from 8-10 wells per genotype. Morphology: n=10-13 neurons imaged from 4-6 wells per genotype. For all panels, control: black circles, G118E: red squares and G118E-E2KO: blue triangles. Statistical analyses of panels C and I were performed by mixed effects model with multiple comparisons (*in panels C and I indicates statistically significant differences between G118E vs the other two genotypes) and for the other electrophysiology and morphology properties using ordinary one-way ANOVA with Tukey’s multiple comparisons (ns: p>0.05, * p<0.05, ** p<0.01, ***p<0.001). Data are presented as mean±sem, Error bars represent sem. Individual datapoints represent individual neurons.

Patients with RTT display substantial electrophysiological abnormalities(41), including seizures of varying severity. Extensive studies on iPSC-derived neuron models of RTT and *MECP2* deletion have established physiological hallmarks of the disorder including decreased frequency of spontaneous postsynaptic currents(42), increased input resistance, and impaired action potential generation(43). To determine the effect of G118E mutation on the electrophysiological function of iNeurons and to assess whether upregulating mutant MeCP2 normalizes these phenotypes, we measured active and passive membrane properties of G118E, G118E-E2KO, and control neurons. In the NGN2-iNs, we found that the G118E neurons had significantly reduced spontaneous synaptic charge transfer (charge, a measure of spontaneous synaptic currents) compared to control neurons (Fig. 5A, p=0.0120) and this deficit was rescued in the G118E-E2KO neurons. The G118E neurons displayed increased firing rate compared to control neurons (Fig. 5B, C), indicative of higher excitability(4). This hyperexcitability was corrected in the G118E-E2KO neurons. The G118E neurons did not show significant deficits in passive membrane properties such as membrane capacitance (Fig. 5D), membrane resistance (Fig. 5E) and resting membrane potential (RMP) (Fig. 5F). However, the G118E-E2KO neurons displayed significantly higher capacitance (Fig. 5D, p<0.01) and lower membrane resistance (Fig. 5E, p<0.01) than the G118E and control neurons, as well as a more negative RMP than the G118E neurons (Fig. 5F). G118E NPC-iNs showed deficits in evoked action potential firing, and membrane capacitance that were rescued in the G118E-E2KO NPC-iNs. No significant differences in membrane resistance and RMP were found across genotypes (Fig. S9A–F). Our data indicate that the G118E mutation causes electrophysiological deficits and that these deficits are rescued by upregulating mutant MeCP2 in these neurons by isoform switching. Examining the electrophysiological properties of T158M-WT, T158M-MU, and T158M-E2KO neurons, we found that the T158M-MU neurons only displayed a more negative RMP compared to the T158M-WT neurons and this did not change significantly upon exon 2 deletion (Fig. S10).

Neuronal morphology defects like reduced dendritic arborization have been observed in neurons from post-mortem RTT brains(44). RTT iPSC-derived neuron models display morphological changes including reduction in soma size, dendritic spine density and dendritic arborization(42, 43). Examining the neuronal morphology of G118E, G118E-E2KO, and control NGN2- and NPC-iNs, we found no significant differences in the soma size (Fig. 5G, Fig S9G) and total neurite length (Fig. 5H, Fig S9H) of G118E and G118E-E2KO neurons compared to control neurons. Next, we performed Sholl analysis and found that the G118E neurons have reduced complexity in dendritic arborization compared to control neurons and that this defect was ameliorated in G118E-E2KO neurons (Fig. 5I, Fig. S9I).

Our studies show that upregulating mutant MeCP2 in RTT-G118E neurons by exon 2 deletion ameliorates electrophysiological and morphological deficits, demonstrating the therapeutic potential of *MECP2* isoform switching for RTT.

### An exon-skipping morpholino switches isoforms and upregulates MeCP2

**Fig. 6.**
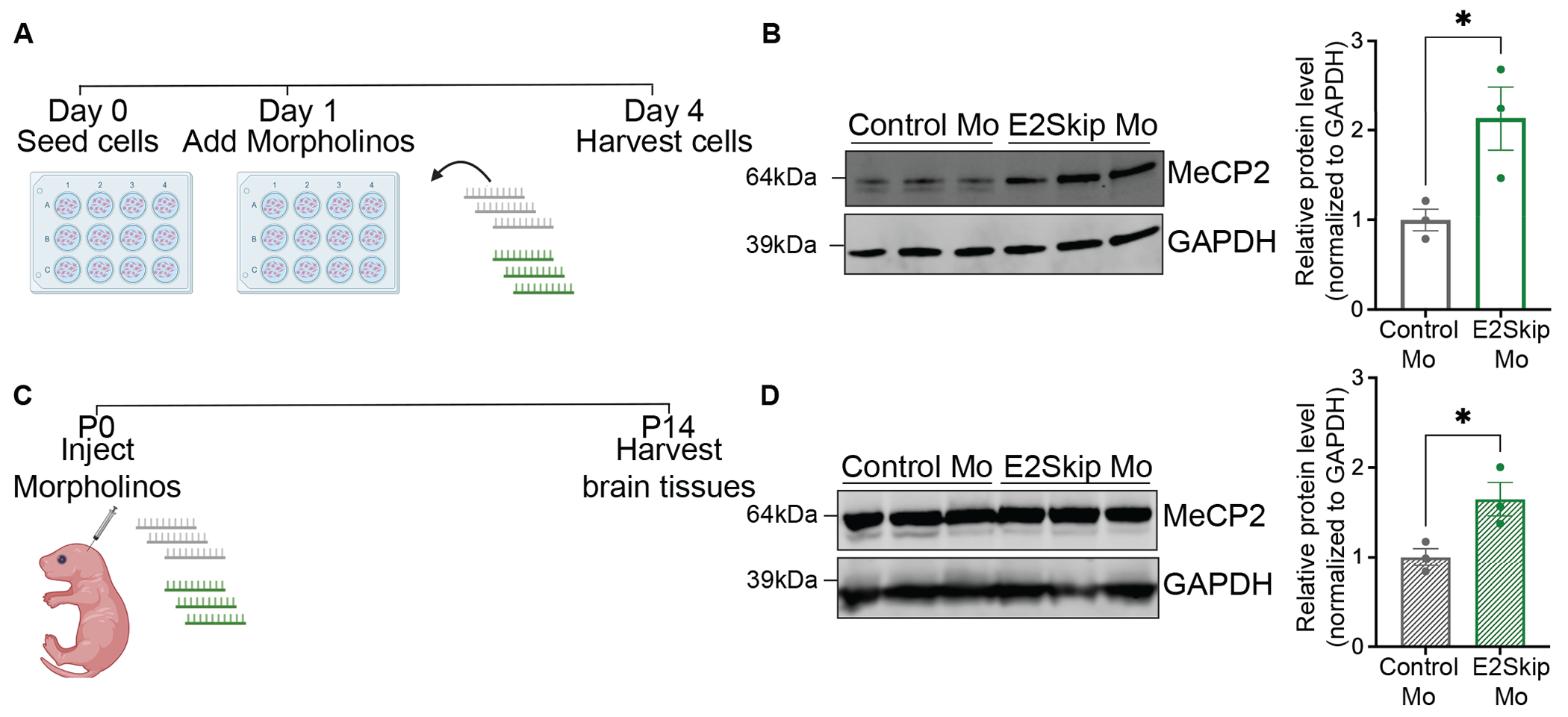
An exon 2-skipping morpholino upregulates MeCP2 protein. (A) Schematic of Morpholino treatment paradigm in HEK293T cells (created with Biorender.com) (B) Left - Western blot showing bands for MeCP2 and GAPDH (internal control) in HEK293T cells treated with Control or E2Skip Morpholino (N=3 each), right - quantification of MeCP2 normalized to GAPDH in these cells (C) Schematic of Morpholino treatment paradigm in P0 FVB wild-type mouse cortices (created with Biorender.com) (D) Left - Western blot bands showing MeCP2 and GAPDH in wild-type FVB male mice cortices injected at P0 with Control Mo or E2Skip Mo (n=3 each) and harvested 2 weeks post-injection, right - quantification of MeCP2 normalized to GAPDH in these tissues. Statistical analyses were done using unpaired t-tests (*p<0.05). Data are presented as mean±sem. Error bars represent sem. Individual datapoints represent biological replicates.

To explore if isoform switching has the potential to be translated into therapy, we designed an exon-skipping morpholino (Mo) to exclude exon 2 from the mature *MECP2* mRNA transcripts. Morpholinos are single stranded, short chains of 20-30 modified nucleic acids that are functionally similar to antisense oligonucleotides (ASOs) and have been widely used for splice switching and steric blocking on target pre-mRNA(45). Testing an exon 2-skipping Morpholino (E2Skip Mo) in HEK293T cells (Fig. 6A), we found that it significantly increased MeCP2 compared to a Control Mo (Fig. 6B, p=0.0395). Since the E2Skip Mo binds to a conserved region in mice, we injected it into wild-type postnatal day 0 (P0) mouse brains (Fig. 6C) and 2 weeks post-injection, saw a significant upregulation of MeCP2 protein in cortices relative to Control Mo-treated mice (Fig. 6D, p=0.0311). These results, combined with the phenotypic improvement of RTT neurons in our genetic deletion model, demonstrate that exon 2-skipping is a promising therapeutic strategy for RTT cases with partially functional *MECP2* alleles.

## DISCUSSION

In this study, we demonstrated that MeCP2 protein abundance can be enhanced by modulating the alternative splicing of *MECP2*. A genetic deletion of exon 2 was effective at upregulating wild-type MeCP2-E1 *in vivo* and most importantly, at upregulating mutant MeCP2-E1 in RTT iNeurons (RTT-iNs). We first tested this strategy in male RTT-iNs harboring a missense mutation p.G118E in the methyl-C binding domain (MBD) of MeCP2 as the male genetic background would allow us to precisely quantify the extent of mutant MeCP2 upregulation achieved by isoform switching and assess the functional consequences of this upregulation in a clean RTT context, without the confounding effect of X-inactivation. The G118E mutation is severe enough to cause premature death during childhood for the affected individual. Importantly, this mutation leads to both reduced MeCP2 protein and reduced DNA binding. Mice carrying this mutation recapitulate majority of the RTT behavioral phenotypes including reduced survival(4). G118E RTT-iNs display reduced MeCP2 compared to the isogenic control lines and exhibit robust transcriptomic, electrophysiological, and morphological phenotypes, establishing both the face and construct validity. We showed that isoform switching increases endogenous mutant MeCP2 to be similar to control and improves transcriptomic dysregulation, electrophysiological and morphological deficits in these G118E neurons. To test whether this isoform switching strategy can benefit severe RTT mutations commonly occurring in female patients with RTT, we deleted exon 2 in a female iPSC-derived neuron model carrying the T158M mutation that drastically reduces MeCP2 by ~70% and impairs DNA-binding ability of MeCP2(10). Isoform switching in the T158M iNs led to a mild upregulation of MeCP2 but importantly, it led to at least a partial rescue of 66.4% of the T158M disease genes with 45.9% of them being rescued by more than 50% magnitude. Although this is a more modest rescue compared to the G118E neurons, given that the T158M-MU neurons have very low MeCP2 to begin with, it is remarkable that isoform switching, even with a small increase in MeCP2 resulted in a dramatic transcriptomic rescue. Our findings are in line with previous studies that showed overexpression of the mutant (T158M)-MeCP2 *in vitro* and *in vivo* resulted in increased binding of the mutant MeCP2 to DNA(9). This marked improvement in transcriptomic disease signature suggests that even a small increase of MeCP2 is functionally impactful in the context of severe RTT mutations. Our work provides proof-of-concept data to support the use of isoform switching as a therapeutic strategy to enhance MeCP2 abundance.

*MECP2* is a dosage-sensitive gene whose loss-of-function and overexpression are both associated with disease. Therefore, any therapeutic strategy involving replacement or upregulation of MeCP2 in patients with RTT would have to be strictly controlled and modulated throughout life to prevent MDS-like symptoms due to overtreatment. Currently, the only FDA-approved treatment for RTT is Trofenitide whose exact mechanism of action to treat RTT is not fully understood(46). There are two gene therapies in clinical trials, both involving AAV-mediated one-time delivery of healthy *MECP2* that is designed to be auto-regulated(13, 14). Our proposed isoform switching approach has several potential advantages for treating RTT. First, we demonstrated that a moderate upregulation of endogenous MeCP2 is sufficient to rescue the functional consequences of a hypomorphic allele. Second, we showed that moderate upregulation of wild-type MeCP2 in ~50% of cells in female mice does not lead to behavioral deficits. Third, using a severe, common RTT mutation we showed that even minimal upregulation of a severely impaired protein can confer functional benefit. Fourth, the isoform-switching approach can be clinically implemented using splice-switching ASOs to skip exon 2 of *MECP2* in the brain. ASO delivery intrathecally has been shown to achieve broad biodistribution in the brain(47) which could prove favorable until gene therapy technologies advance to overcome the localized delivery and limited biodistribution in the brain(15, 16).

Our study has limitations. A limitation of the isoform switching approach is that it requires patients to produce at least a partially functional protein. For mutations that eliminate binding or are true nulls due to early truncation or deletion, this approach will not work. Fortunately, this approach could help ~ 50% of patients with partially functional *MECP2* alleles (RettBase)(11). Here, we demonstrated functional improvements achieved by isoform switching across *MECP2* mutations spanning mild (G118E) to severe (T158M) disease severity. We showed that an exon 2 skipping morpholino upregulated MeCP2 protein *in vivo* in the mouse brain by ~60%. However, we limited our assessment to MeCP2 protein quantification since morpholinos (both control and E2Skip) caused long-term toxicity (hydrocephalus) in the mice, a phenomenon previously reported by other studies using morpholinos(48, 49). Future work with an exon 2-skipping ASO examining the benefits of isoform switching across RTT mutations that lead to varying degrees of MeCP2 reduction could determine the extent of phenotypic improvements in a mutation-specific manner.

To date, there are several ASOs approved by the FDA to treat disorders like spinal muscular atrophy (SMA) and Duchenne muscular dystrophy(50). The ASO for SMA results in increasing full length SMN protein by exon inclusion and the ASOs for DMD work by exon skipping of the pathogenic mutation-containing exon. Our proposed mechanism of *MECP2* exon 2-skipping to facilitate isoform switching for RTT treatment uses an approach similar to ASOs targeting pre-mRNA splicing but is unique as it increases the translationally efficient mRNA isoform and results in modest upregulation of endogenous protein. Our data using an exon-skipping Morpholino to exclude *MECP2* exon 2 and upregulate MeCP2 protein in human cells and mouse brain are encouraging.

In conclusion, our work lays the foundation and provides preclinical evidence for an ASO-based therapeutic approach for RTT that upregulates MeCP2 and confers functional improvement. We envision the isoform switching strategy to be potentially applied to other disorders caused by loss of function of protein wherein the gene encodes alternatively spliced isoforms with different translational efficiencies to upregulate target protein for therapeutics.

## MATERIALS AND METHODS

### Study design

The objective of this study was to determine whether switching *MECP2-e2* to *e1* would upregulate endogenous MeCP2 protein and ameliorate RTT phenotypes. We first checked *e1* and *e2* mRNA and protein abundance in post-mortem human PFC tissues. Next, we generated a *Mecp2* E2KO mouse model to assess effects of *e2* depletion on MeCP2 protein and neurological function. Mouse maintenance, breeding and experimental use protocols were approved by Baylor College of Medicine Institutional Animal Care and Use Committee (IACUC, Protocol AN-1013). The use of human iPSC lines was approved by the Baylor College of Medicine Institutional Review Board (IRB protocol # H-34578). We deleted exon 2 in two human RTT patient-derived iPSC lines (RTT-E2KO iPSCs). We differentiated two clones each of the G118E, isogenic control and G118E-E2KO iPSCs into neurons by two methods: NGN2-overexpression and NPC-differentiation. We differentiated one clone each of the T158M-MU, T158M-WT and T158M-E2KO iPSCs by NGN2-overexpression. We measured MeCP2 protein in the iNeurons and to assess effect of exon 2 deletion on RTT phenotypes, we performed bulk RNA-sequencing, electrophysiology and morphology analyses. Lastly, we tested effect of an exon 2-skipping morpholino on MeCP2 protein abundance in HEK293T cells and P0 mouse brain by ICV injection. We determined sample size using power calculations based on previously published papers and according to field standards. Replicate numbers and statistical tests used are given in the figure legends. All western blots, qRT-PCRs and Morpholino experiments were performed at least twice (independent replicates). For all experiments, outliers were excluded based on outlier analysis. The experimenter was blinded to genotype for mouse behavior, electrophysiology and morphology experiments in iNeurons.

### Human postmortem tissue samples

Post-mortem PFC tissues from control individuals provided by Parkinson’s UK Brain Bank were used to measure *MECP2-e1* and *e2* mRNA and protein isoforms as described below. See Table S1 for demographic details of individuals.

### Exon 2 deletion (E2KO) mice generation

*Mecp2* exon 2 deletion was generated by CRISPR/Cas9-mediated gene editing. gRNAs were designed per guidelines outlined by (51) (Table S2) and editing was performed as previously described(52). Details in supplementary methods.

### Mouse breeding and colony maintenance

Mice were housed in AALAS-certified level-3 facility on a 14-hour light cycle and monitored daily. E2KO mice were maintained on a C57BL6/J background. CFW pregnant females from Charles River were used for P0 injections.

### Protein extraction and Western blot

Mouse protein lysates were prepared as described previously(35). Postmortem PFC tissue and adherent cell lysate preparation is described in supplementary methods. Western blots for all samples except PFC tissue were performed as described previously(35). We used the anti-MeCP2 antibody that detects both isoformsx(53) (Cell Signaling Technology #3456, 1:1000). Primary antibodies and dilutions for internal control proteins: anti-vinculin (Sigma-Aldrich #V9131; 1:5000) and anti-GAPDH (Advanced ImmunoChemical Inc. #2-RGM2, 1:10000). Secondary antibodies for mouse tissues and cell culture samples: LI-COR Biosciences #926-32211 and # 926-68070, both 1:2000). For postmortem PFC tissues, detection was done with HRP-conjugated secondary antibodies and ECL reagents (Cytiva, # RPN2232)(54). Raw blot images are provided in supplementary data file S1.

### RNA extraction, cDNA synthesis and quantitative PCR

RNA extraction was performed per manufacturer guidelines using RNeasy Mini kit (Qiagen, #74106). cDNA was synthesized from 500 ng-1 μg RNA using PrimeScript^™^ RT Master Mix (Takara, # RR036B). Quantitative PCR (qPCR) was performed using PowerUp^™^ SYBR^™^ Green Master Mix (ThermoFisher, #A25743) on CFX384 Real-Time PCR System (Bio-Rad). Relative gene expression was calculated by ΔΔCt. Standard curve qPCR was performed as described in supplementary methods. Primer sequences in Table S3. All reactions were performed with at least three biological replicates and at least two technical replicates.

### Behavioral analyses

Mice were habituated for at least 30 minutes before each test. For male E2KO mice and wild-type littermates, open field assay and rotarod assay were performed on consecutive days. Two days after this, elevated plus maze was performed at 10-11 weeks. These animals were aged to 6 months, and contextual and cued fear conditioning assay was performed. For heterozygous female E2KO mice and wild-type littermates, open field assay was performed followed by elevated plus maze at 15-16 weeks. Assays were performed as previously described(33) and detailed in supplementary methods.

### Genome editing of RTT iPSCs

G118E and paired isogenic control iPSCs (male) have been described previously(4). T158M and paired isogenic control iPSCs (female) were obtained from the Coriell Institute for Medical Research under an MTA with Rett Syndrome Research Trust. To delete *MECP2* exon 2 in the iPSCs, sgRNAs were designed per previously outlined guidelines(51) (Table S2). CRISPR/Cas9 editing in the G118E and T158M-MU iPSCs was performed as described in supplementary methods. Infected iPSC colonies were screened by genomic PCR using primers in Table S3. iPSCs were tested for mycoplasma. Two clones each for G118E, G118E-E2KO and control, and one clone each for T158M, T158M-E2KO and control were used for our studies.

### Culturing, maintenance, and storage of iPSCs

iPSCs were cultured and maintained under feeder-free conditions using Matrigel and mTeSR plus media (Stemcell Technologies, # 100-1130) with 1% Penicillin-Streptomycin (Sigma-Aldrich, # P0781). iPSCs were passaged using ReLeSR (Stemcell Technologies, #100-0483) and cryopreserved using Bambanker Serum-Free Cell Freezing Medium (Wako Chemicals, #302-14681).

### NGN2-driven neuronal induction and differentiation of iPSCs

For G118E, G118E-E2KO and control iPSCs, rtTA and hNGN2 lentivirus were prepared(4) and iPSCs were infected sequentially with rtTA lentivirus ( Addgene #66810), and hNGN2 lentivirus (Addgene #79823) with appropriate selection for each construct. For the T158M, T158M-E2KO and control iPSCs, a single construct with rtTA and NGN2 allowing faster transduction (Addgene #127288) was used to prepare lentivirus and iPSCs were infected at MOI 5. We only used the NGN2-overexpression method for the T158M line as it allows faster, efficient neuronal transduction, results in more mature neurons earlier compared to the NPC-derived neuronal differentiation method and overall, the disease phenotypes and rescue by E2KO in the G118E iNs were comparable between both methods of differentiation. Puromycin selection was done(55). Infected iPSCs were expanded and plated for neuronal differentiation as described in supplementary methods.

### Generation of neural progenitor cells (NPCs) from iPSCs and differentiation into neurons

NPCs were derived using a variation of dual SMAD inhibition protocol(56, 57). Details of NPC generation and differentiation are provided in supplementary methods.

### Immunofluorescence studies

Immunofluorescence studies on iNeurons were performed as described previously(34). Imaging was done on Leica SP8 confocal microscope. Antibodies and dilutions in Table S4.

### Bulk RNA-sequencing

1 μg RNA was sent to Azenta LifeSciences for bulk RNA sequencing at ~40 million reads per sample on NovaSeq6000. FASTQ files quality was checked using FASTQC, reads were trimmed using Trimmomatic v0.39 with default parameters (ILLUMINACLIP:TruSeq3-PE.fa:2:30:10:2)(58). Trimmed sequences quality was checked with FastQC and reads aligned to GRCh38p12 primary assembly, v28 from GENCODE using STAR v2.7.9a(59), with default parameters and alignment quality was checked with RSeQC(60). DESeq2 was used to generate normalized gene expression values (61). Downstream analyses are described in supplementary methods. DEG lists of G118E NGN2-iNs (supplementary data file S2), G118E NPC-iNs (supplementary data file S3) and T158M NGN2-iNs (supplementary data file S4) are provided as supplementary material.

### Electrophysiological studies

Electrophysiology was done by whole cell patch clamp method. Recording protocol was adapted and modified from previous work(62, 63). Details are described in supplementary methods.

### Neuronal morphology analysis

NGN2- and NPC-iNs were prepared as described in supplementary methods. Neurons were imaged on Zeiss LSM 710 confocal microscope. Tracings were done on the Neurolucida software using the user-guided setting and soma area was measured on Image J.

### Morpholino synthesis and sequence

Control and E2Skip morpholinos were synthesized by Gene Tools LLC by coupling one base at a time using optimized chemistry. Synthesized oligos underwent selective precipitation to remove waste products (synthetic resin, ammonia, cleaved base-protective groups, and minor amounts of short truncation fragments). They performed spectrophotometric quantitation and MALDI-TOF spectral analysis, following which the morpholinos were freeze dried, sterilized and shipped. Once received, the morpholinos were resuspended in an appropriate volume of sterile 1x DPBS for use. The control Morpholino used in our studies is the Gene Tools Vivo Morpholino Standard Control oligo. The sequence of the E2Skip Morpholino is:

AAGGAAGGTTACTTACCTGAGCCCT

### Treating HEK293T cells with Morpholino

HEK293T cells were plated at 100,000 cells/well in a 24-well plate in antibiotic-free DMEM [+] 4.5 g/L glucose, L-glutamine, sodium pyruvate (Corning, #10-013-CV) with 10% Fetal Bovine Serum (R&D Systems, #S11150) and incubated at 37 °C, 5% CO_2_ overnight. Mo (Control or E2Skip) was diluted in 250 μL of antibiotic-free culture medium and 250 μL of the medium in each well was replaced with Mo-containing medium for a concentration of 10 μM. Cells were incubated with Mo for 72 hours at 37 °C, 5% CO_2_ and were harvested for protein extraction.

### P0 injections with Morpholino

P0 neonatal mice (<12 hours post-birth) from timed-pregnant CFW Swiss Webster female were anesthetized on ice. 2 μL E2Skip or Control Mo was injected (at 2.5 ng/μL) into each lateral ventricle as described previously(64). Pups were monitored daily for 3 days post-injection and then bi-weekly until harvest of brain tissues.

### Statistical analyses

Statistical analyses of biochemical, behavior, electrophysiology and neuronal morphology data were done using GraphPad Prism and for RNA-sequencing data on R. Parametric or nonparametric tests were used based on normality tests of data. Unless otherwise indicated, data are represented as mean±SEM. Number of biological and technical replicates, specific statistical tests used for each experiment and significance statistics are in the figure legends. Individual-level data for all experiments with sample size n<20 has been provided in supplementary data file S5.

## Supplementary Material

MDAR checklist_Tirumala et al

Supplementary data file S3

Supplementary data file S4

Supplementary data file_S5

Supplementary data file_s2

Supplementary data file S1

Supplementary materials_Tirumala et al

## Data Availability

All data associated with this study are present in the paper or supplementary materials. All materials newly generated in this study (E2KO mouse line, two G118E-E2KO iPSC clones and one G118E iPSC clone) will be available upon request from the corresponding author under a Material Transfer Agreement with Baylor College of Medicine. The T158M-MU-E2KO iPSC line which will be deposited at RSRT. The T158M-WT and MU iPSCs used in this study were provided by RSRT under an MTA. All other materials used or generated in this study are commercially available or will be supplied upon reasonable request. The data presented in this study are deposited on GEO (GSE268177). RNA-sequencing analysis code is available on Zenodo (DOI: 10.5281/zenodo.18140370).
