# Supplementary material for: Modulating alternative splicing of MECP2 is a potential therapeutic strategy for Rett syndrome
Source: Sci Transl Med. Author manuscript; Available in PMC 2026 Apr 8. (PMC13061089; doi:10.1126/scitranslmed.adq4529)

**Supplementary File**

**Raw Western Blot Images**

Below are the raw Western blot images of all the blots shown in main and supplementary figures in the manuscript.

Figure 1B– Human Prefrontal Cortex postmortem samples Western blot raw images:


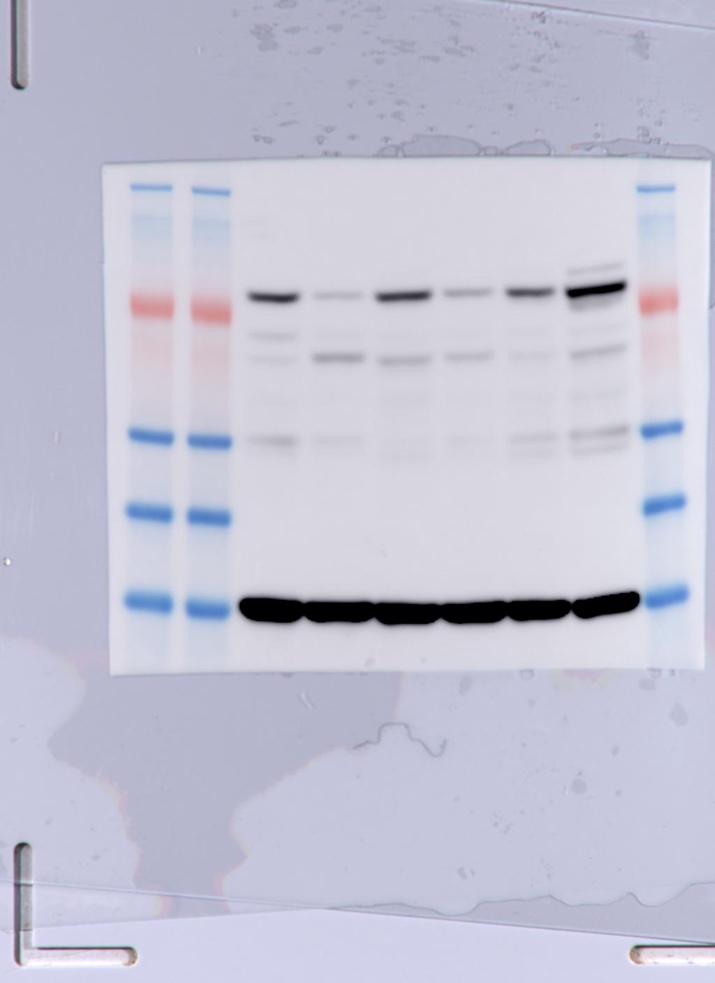


39kDa (GAPDH)

Image 1. Western blot image of human prefrontal cortex samples (Lanes left to right: Ladder, PDC 022, PDC 029, PDC 023, PDC 026, PDC 040, Ladder, Ladder). This image was captured after 10 seconds of exposure time and used for quantification of GAPDH bands (labelled with arrow). *Sample PDC023 was excluded from quantification analysis and from Figure 1C.


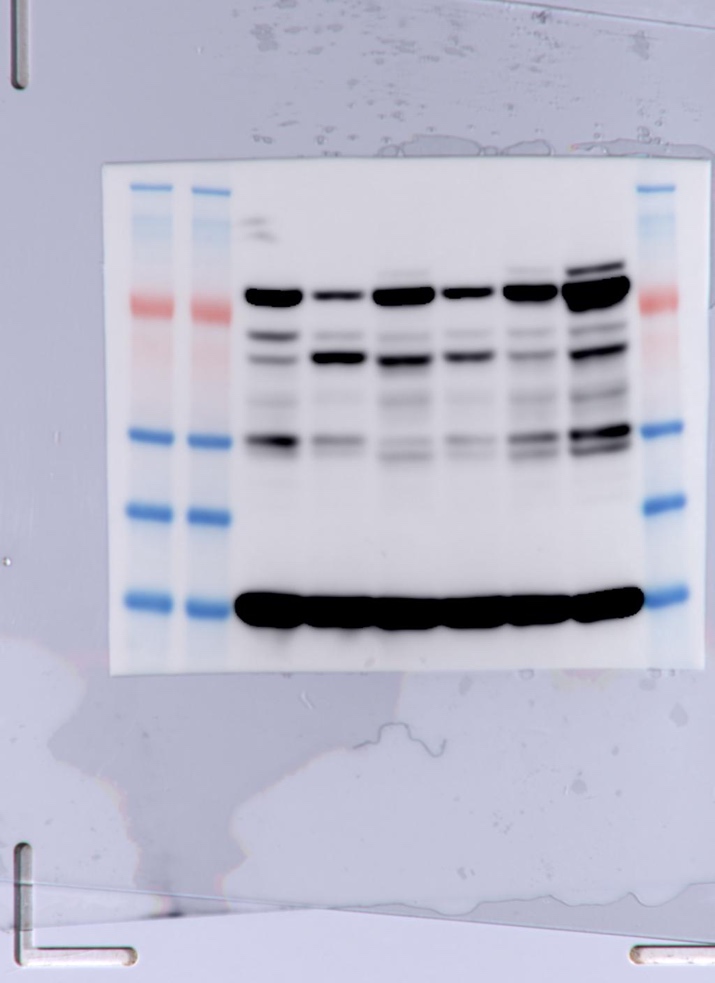


64kDa

(MeCP2)

39kDa

(GAPDH)

Image 2. Western blot image of human prefrontal cortex samples (the same blot as above) was captured after 40 seconds of exposure time to visualize MeCP2 bands (labelled with arrow) and used for quantification of MeCP2 bands. *Sample PDC023 was excluded from quantification analysis and from Figure 1C.

Figure 2A – WT and E2KO mice cortices Western blot raw image:


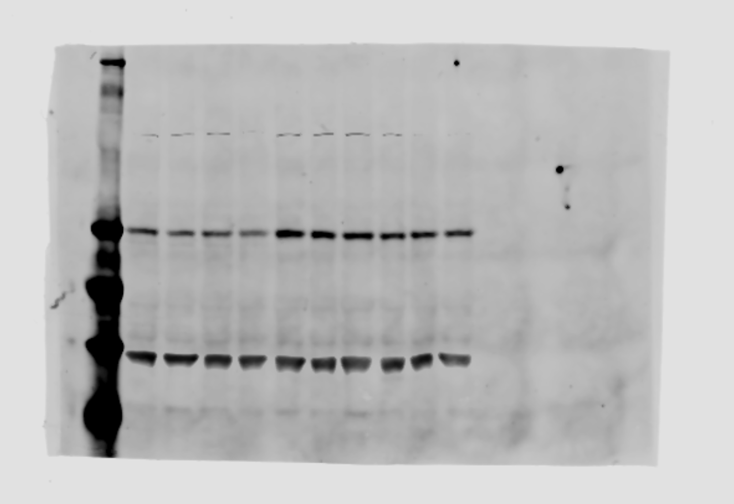


39kDa

(GAPDH)

64kDa

(MeCP2)

Image 3. Western blot image of wild-type (WT) and exon 2 knockout (E2KO) mice cortex samples showing bands for MeCP2 and GAPDH (labelled with arrows). Lanes (left to right): Ladder, WT samples (N=4), E2KO samples (N=6).

Figure 3D – Isogenic control, G118E, G118E-E2KO NGN2-iNs Western blot raw image:


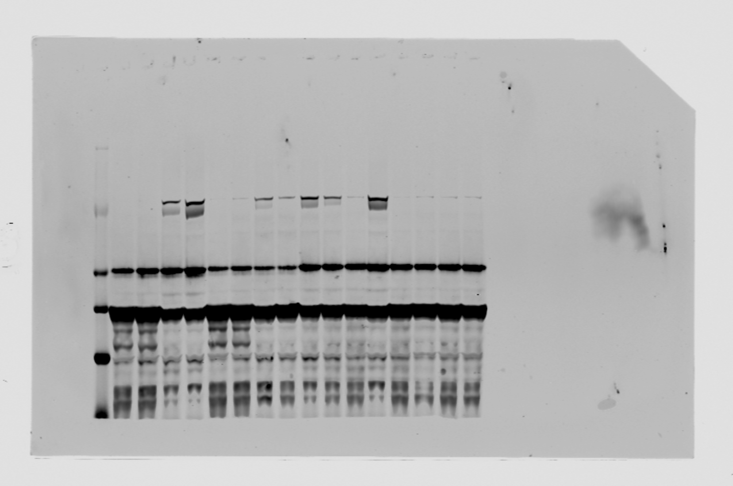


64kDa

(MeCP2)

39kDa

(GAPDH)

Image 4. Western blot image of isogenic control, G118E and G118E-E2KO NGN2-iNeuron samples showing bands for MeCP2 and GAPDH (labelled with arrows). Lanes (left to right): Ladder, isogenic control (n=4), G118E (n=4) and G118E-E2KO (n=8). Only the first 4 G118E-E2KO samples were included in the main figure 3D and in the quantification analysis.

Figure 6B – HEK293T cells treated with Control Morpholino and E2Skip Morpholino Western blot raw image:


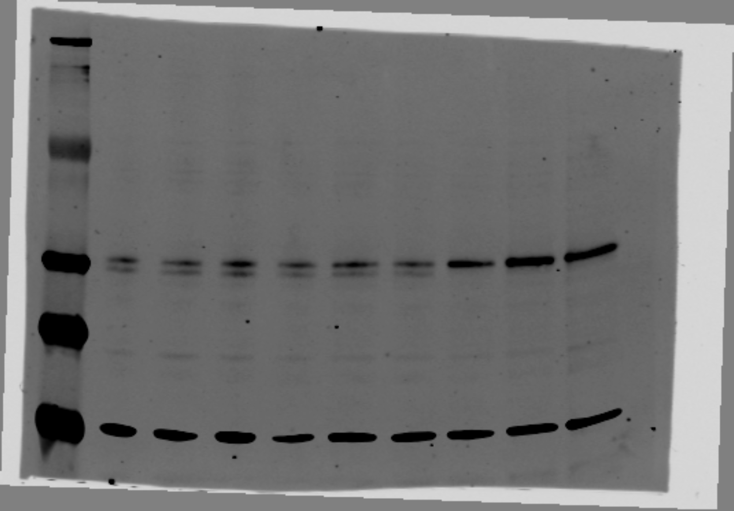


64kDa

(MeCP2)

39kDa

(GAPDH)

Image 5. Western blot image of HEK293T cells treated with Control Morpholino or E2Skip Morpholino showing bands for MeCP2 and GAPDH (labelled with arrows). Lanes (left to right): Ladder, Untreated HEK293T samples (n=3), Control Morpholino-treated HEK293T samples (n=3) and E2Skip Morpholino-treated HEK293T samples (n=3). The 3 untreated HEK293T samples were not included in the main figure and the quantification analysis.

Figure 6D – Wild-type mice cortices treated with Control Morpholino and E2Skip Morpholino Western blot raw image:


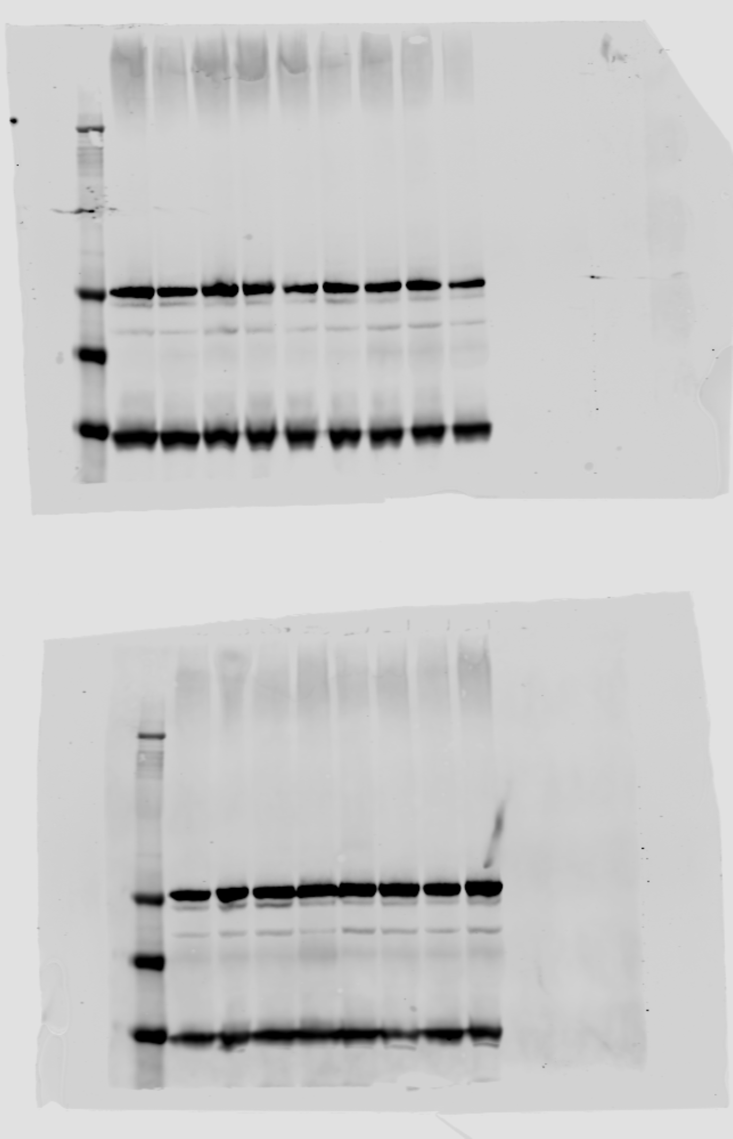


39kDa

(GAPDH)

64kDa

(MeCP2)

Image 6. Western blot image of 2-week-old wild-type mice cortices treated at P0 for 2 weeks with Control Morpholino or E2Skip Morpholino showing bands for MeCP2 and GAPDH (labelled with arrows). Lanes (left to right): Ladder, Control Morpholino-treated mouse cortex samples (n=4) and E2Skip Morpholino-treated mouse cortex samples (n=4). The first Control Morpholino-treated sample and the last E2Skip Morpholino-treated sample were not included in the main figure and in the quantification analysis.

Figure S1A – WT and E2KO mice hippocampus samples Western blot raw image:


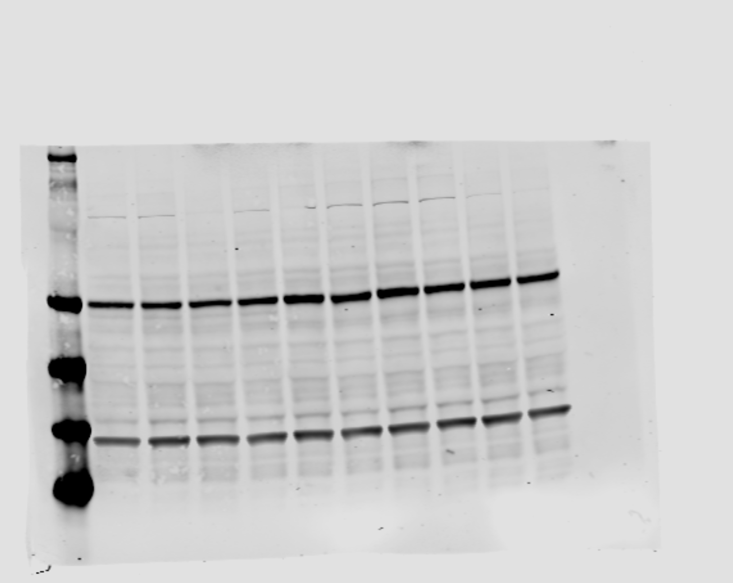


64kDa

(MeCP2)

39kDa

(GAPDH)

Image 7. Western blot image of wild-type (WT) and exon 2 knockout (E2KO) mice hippocampus samples showing bands for MeCP2 and GAPDH (labelled with arrows). Lanes (left to right): Ladder, WT samples (N=4), E2KO samples (N=6).

Figure S1B – WT and E2KO mice brainstem samples Western blot raw image:


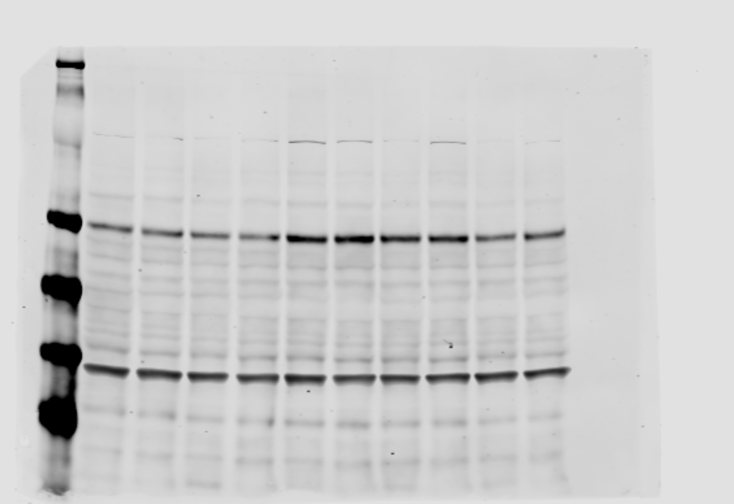

Image 8. Western blot image of wild-type (WT) and exon 2 knockout (E2KO) mice brainstem samples showing bands for MeCP2 and GAPDH (labelled with arrows). Lanes (left to right): Ladder, WT samples (N=4), E2KO samples (N=6).

64kDa

(MeCP2)

39kDa

(GAPDH)

Figure S1C – WT and E2KO mice cerebellum samples Western blot raw image:


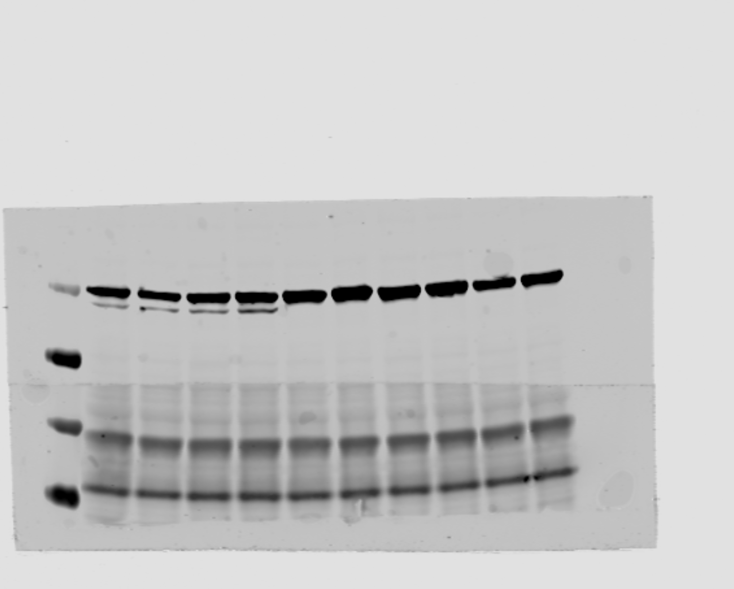


64kDa

(MeCP2)

39kDa

(GAPDH)

Image 9. Western blot image of wild-type (WT) and exon 2 knockout (E2KO) mice cerebellum samples showing bands for MeCP2 and GAPDH (labelled with arrows). Lanes (left to right): Ladder, WT samples (N=4), E2KO samples (N=6). The line above the GAPDH bands in the blot is because this western blot was cut in between the MeCP2 and GAPDH bands in order to incubate with separate primary antibodies (MeCP2 and GAPDH).

Figure S3D – Isogenic control, G118E, G118E-E2KO NPC-iNeurons Western blot raw image:


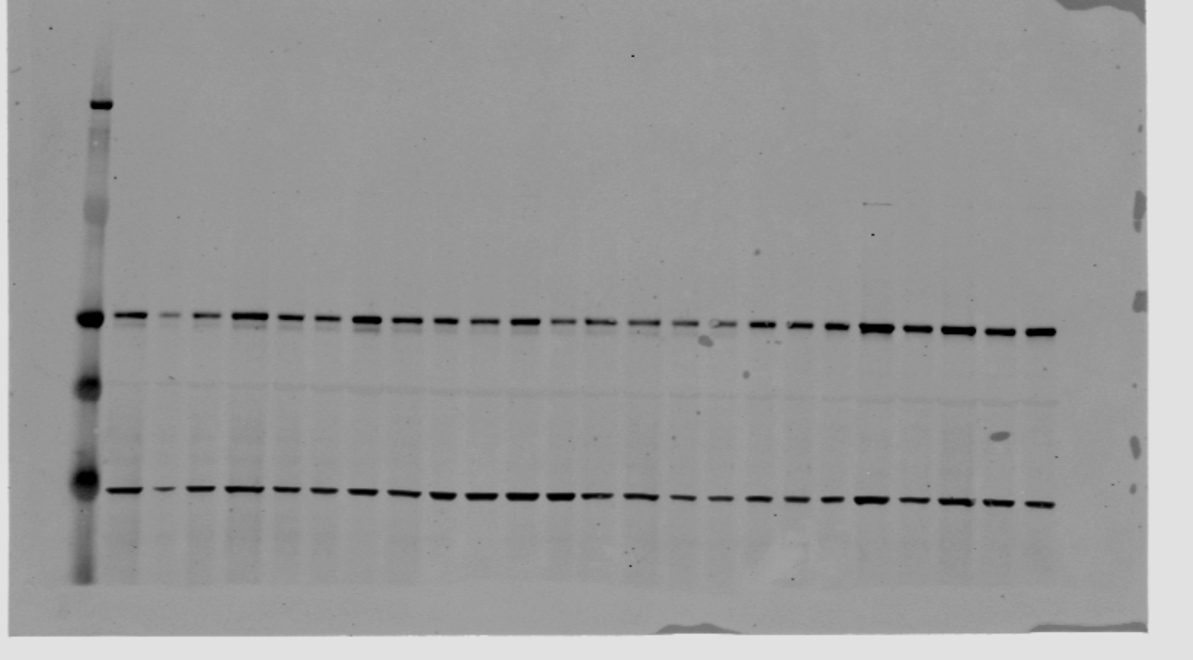


39kDa

(GAPDH)

64kDa

(MeCP2)

Image 10. Western blot image of isogenic control, G118E and G118E-E2KO NPC-derived iNeuron samples showing bands for MeCP2 and GAPDH (labelled with arrows). Lanes (left to right): Ladder, isogenic control (n=8, two clones n=4 each), G118E (n=8, two clones n=4 each) and G118E-E2KO (n=8, two clones n=4 each).

Figure S4C – T158M-WT, T158M-MU and T158M-MU-E2KO NGN2-iNeurons Western blot raw images (11-12):


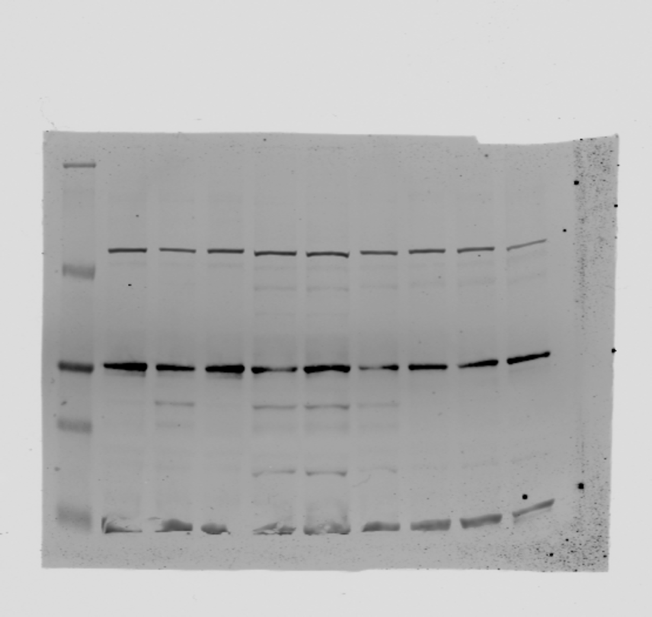


64kDa

(MeCP2)

97kDa

(Vinculin)

Image 11. Western blot image of T158M-WT, T158M-MU and T158M- E2KO NGN2-iNeuron samples showing bands for MeCP2 and Vinculin (labelled with arrows). Lanes (left to right): Ladder, T158M-WT (n=3), T158M-MU (n=3) and T158M-E2KO (n=3).


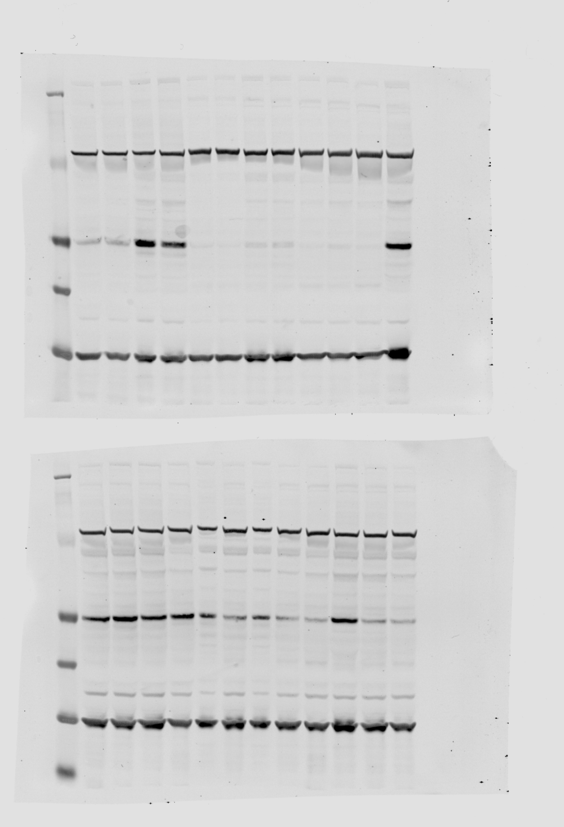


64kDa

(MeCP2)

97kDa

(Vinculin)

64kDa

(MeCP2)

97kDa

(Vinculin)

Image 12. Western blot images of T158M-WT, T158M-MU and T158M-MU-E2KO NGN2-iNeuron samples showing bands for MeCP2 and Vinculin (labelled with arrows). Top blot and bottom blot lanes (left to right): Ladder, T158M-WT (n=4), T158M-MU (n=4) and T158M-E2KO (n=4).

**GTG-banding analysis files of G118E-E2KO iPSCs**

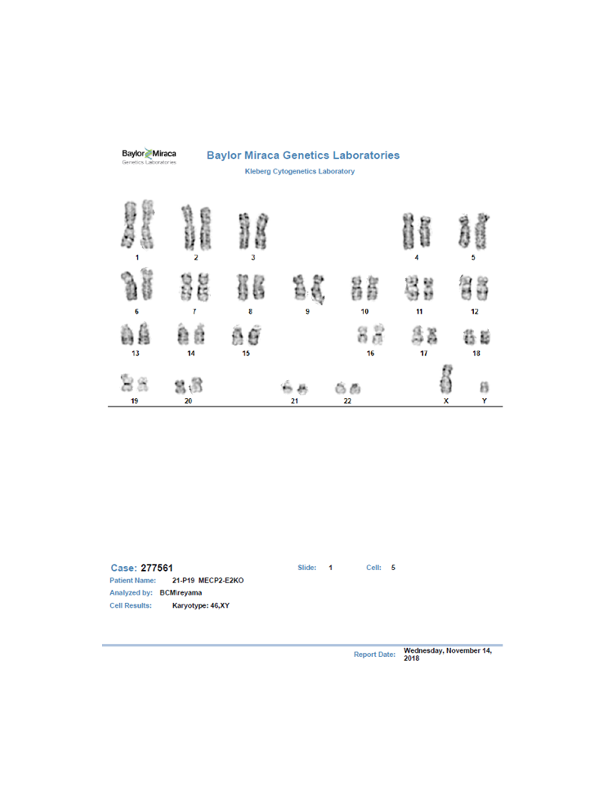

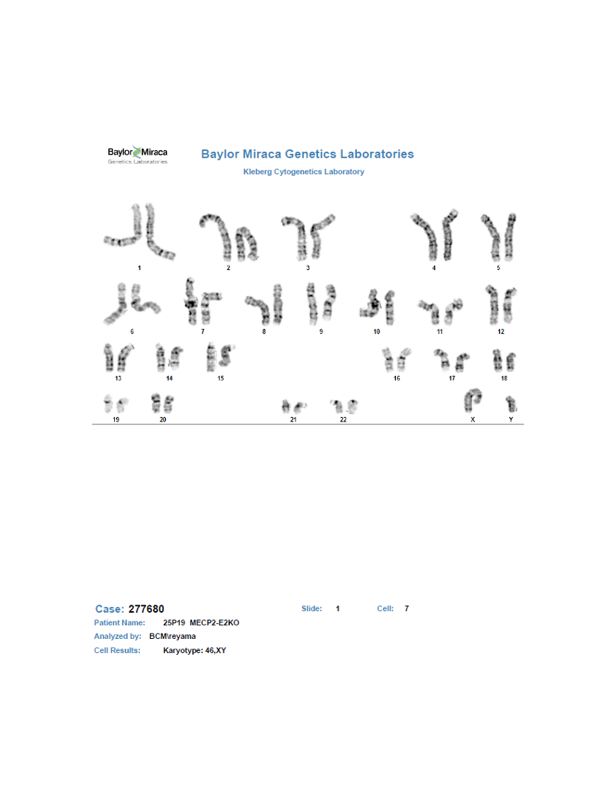

Supplement: Supplementary data file S1 [file NIHMS2154394-supplement-Supplementary_data_file_S1.docx]
