# Supplementary material for: Modulating alternative splicing of MECP2 is a potential therapeutic strategy for Rett syndrome
Source: Sci Transl Med. Author manuscript; Available in PMC 2026 Apr 8. (PMC13061089; doi:10.1126/scitranslmed.adq4529)
Supplement: Supplementary materials_Tirumala et al [file NIHMS2154394-supplement-Supplementary_materials_Tirumala_et_al.pdf]

SUPPLEMENTARY METHODS

Exon 2 deletion mouse generation

Cas9 nuclease (Integrated DNA Technologies, IDT, 1081060), tracrRNA (IDT, 1072532) and sgRNAs were injected (pronuclear) into ova from wild-type C57BL6/J females and transferred into oviducts of pseudo-pregnant females. Founders were sequence-verified by PCR and Sanger sequencing using primers flanking exon 2 (Table S2) and E2KO progeny were backcrossed at least five generations.

Protein extraction and Western blot

Postmortem human tissue was homogenized in 100 µL NP-40 lysis buffer (with 2%SDS, 1x protease inhibitor and 1x phosphatase inhibitor) and protein extraction was performed as described previously*(36)*. For adherent cells, NP-40 lysis buffer was added, cells were scraped and rocked at 4 °C for 15 minutes. Lysis was continued and western blots were done as described previously*(36)* with 10-20 µg of protein per sample. Imaging was done on LI-COR Odyssey CLx Imager and quantification on Image Studio™ Lite software. Western blots for all samples except the human postmortem tissues were performed as described previously*(36)*.

Standard curve quantitative real-time reverse transcription polymerase chain reaction (qRT-PCR)

For standard curve qRT-PCR, PAGE-purified Ultramer™ DNA Oligonucleotides of *e1* and *e2* amplicons were ordered from IDT. Serial dilutions (10 pM-100 aM) of these oligos were used

for all standard curve qPCRs. Absolute mRNA concentration was determined using the straight-line equation. Primers and oligo sequences in Table S3.

Behavioral analyses

The following behavioral assays were performed in exon 2 deletion (E2KO) and littermate control wild-type mice.

Open field assay

Open field assay was performed as described previously*(33)*. Briefly, each mouse was placed in an open field chamber enclosed by four walls (open at the top) and allowed to explore freely for 30 minutes. The open field has laser beams across its length and breadth and the activity of the mouse was measured by the number of beam breaks recorded by the Fusion software and analysis of the locomotion was done using the following parameters: the ratio of distance travelled within the center of the chamber to the total distance travelled, time spent in the center and number of entries to the center. These parameters were analyzed using a one-way ANOVA for each parameter to determine if there is a significant difference across genotypes.

Accelerated Rotarod assay

The assay was setup and performed as previously described*(33)* with slight modifications as described below. Briefly, each mouse was placed on a rotating cylindrical rod that accelerates from 4 RPM to 40 RPM over a duration of 5 minutes and motor coordination was measured based on the time taken for each mouse to either fall off the rotating rod or to spin around the rod twice consecutively without attempting to walk (latency to fall). The maximum time on the rotarod is 5 minutes, after which if the mouse has not fallen, it was removed. This assay was performed 4 times in a day with at least 30 minutes between each trial and the set of 4 trials were

repeated for 4 consecutive days. Repeated measures two-way ANOVA was used to analyze and compare the latency to fall across genotypes.

Elevated plus maze

The assay was set up as described previously*(33)*. Each mouse was placed in the center of an elevated, plus-shaped maze with two closed and two open arms and allowed to explore freely for 10 minutes. Using ANY-maze software, the movement of the mouse was tracked, and the duration spent, and distance travelled by the mouse in the center, open and closed arms of the maze was recorded. One-way ANOVA was used to analyze the differences across genotypes based on the normalized distance (distance in open arm/total distance), time spent in the open arm, closed arm and center of the maze and number of entries to the open arm, closed arm and center.

Contextual and cued fear conditioning

For this assay, the three-step protocol published previously was used*(33)*. For this test, only the mice being immediately tested were brought into the assay room with the other mice left in a holding room to prevent them from being affected by the vocalizations of the subject mice. On the first day (training), each mouse was trained by placing it in a closed chamber, allowed to habituate for 2 minutes, and presented with a sound (80dB) for 30 seconds, with a foot shock (0.7 mA) during the last 2 seconds of the 30-second sound. This pattern was repeated once. 24 hours later, contextual memory was tested followed by cued memory with at least one hour between the two tests. For contextual memory, the environment was kept the same as the training day and the mouse was placed in the chamber for 5 minutes without any sound or foot shock (only context) and the freeze response of the mouse was recorded by a camera fitted in the chamber. For cued memory, changes were made to room lighting, test chamber’s shape, color,

and scent to mimic a new context. The mouse was placed in this chamber and allowed to habituate for 3 minutes following which the sound (same as training day) was played for 3 minutes without foot shock. The freeze response to this paradigm was also recorded by the camera. The Freeze Frame 4 system was used to record freezing bouts, the time and frequency of freezing which were analyzed by one-way ANOVA to determine differences across genotypes.

CRISPR/Cas9 genome editing for exon 2 deletion in iPSCs

G118E and T158M-MU iPSCs were adapted to single-cell passaging using Accutase and StemFlex medium (Thermo Fisher Scientific, #A3349401) and on the day of nucleofection, iPSCs were pre-treated with 10 μM ROCK inhibitor Y-27632 (Tocris #1254) for 30 minutes, dissociated to single cells and 300,000 cells nucleofected with 200 pmol sgRNA (Synthego) complexed to 40 pmol Cas9 protein (Thermo Fisher Scientific, A36496) using 4D-Nucleofector^{TM} X Kit S (Lonza; Program CA-137). Nucleofected cells were seeded on Matrigel-coated plate (Corning #354277) in StemFlex medium with 10 μM Y-27632 for the first 24 hours.

NGN2-iNeurons differentiation

For differentiation, infected iPSCs were plated in neuronal induction media (NIM, see recipe below) with 10 μM Y-27632 and doxycycline (1 μg/mL) for 24 hours. Media was replaced with NIM with puromycin (0.5-1 μg/mL) and doxycycline (1 μg/mL) daily for 3 days. Then, media was replaced with neuronal differentiation media (NDM, see recipe below) and half-media changes of this were done once every 3 days until neurons were harvested*(4)*. NIM and NDM recipes and numbers of infected iPSCs plated for each experiment are given below.

Generation of Neural Progenitor cells (NPCs) and differentiation into neurons

Briefly, $2x10^6$ iPSCs/well were plated in an Aggrewell plate in NIM. Next day, ¾ of medium was replaced with fresh medium and dual SMAD inhibition initiated by adding 10 µM SB-431542 (Tocris #1614) and 0.2 µM LDN 193189 (Tocris #6053). Daily media changes with both inhibitors were performed over next four days. Aggregates were collected on day 5, sieved through a reversible strainer and transferred onto Matrigel-coated plates. From day 6 cultures were switched to neural proliferation medium (NPM, see recipe below). The SMAD inhibitors were in media until day 9 with addition of 10µM cyclopamine (Tocris #1623) between days 6-9. Media was changed daily until rosette-shaped clusters of neural progenitors were harvested between days 12-14. For harvest, cultures were incubated for one hour with Rosette Selection Reagent (RSR) at 37 °C, dislodged rosettes were incubated in wells coated with 0.2% porcine gelatin to allow the non-neural cells to differentially attach*(57)*. Floating fraction was collected after 1 hour, transferred into uncoated flasks and incubated in suspension overnight. Next day, floating NPC spheres were plated onto Matrigel-coated plates and propagated to confluency. From this stage, Accutase was used to prepare single-cell suspension for expansion of NPCs. All cultures were maintained in the presence of 1% penicillin-streptomycin. For differentiation, NPCs were thawed in NPM and plated at 2×10^6 in T-75 flasks in NIM with 20 ng/ml FGF2 (Peprotech, AF-100-18B). Media was replaced with fresh NIM every day until day 4. On day 4, cells were plated in NIM with Y-27632 onto Matrigel-coated plates for all experiments as described in supplementary methods. From day 5, media was switched to NDM with 1 µg/ml Laminin (R&D systems, 3400-010-02) and maintained until harvesting with alternating full- and half-media changes every three days, adding Laminin or Matrigel once every other week.

Media recipes used for NPCs and neuronal differentiation of NGN2-iNeurons

NIM recipe: 50:50 mix of DMEM/F-12:Neurobasal media with 2% B27-supplement, 1% N2-supplement, 2 mM Glutamax; NPM: equal volumes of DMEM/F-12 and Neurobasal media with 1% B27-supplement (Vitamin A-free), 0.5% N2-supplement, 2mM Glutamax, 20ng bFGF/ml, and 20ng EGF/ml; NDM: neurobasal medium with 2% B27, 2 mM Glutamax, 20 ng/ml BDNF, 10 ng/ml NT-3, 10 ng/ml GDNF, 200 μM ascorbic acid and 100 μM db-cAMP.

Plating numbers for NGN2-iNeurons and NPC-iNeurons differentiation

NGN2-iNeurons: Infected iPSCs were plated at 100,000 cells/well of a 24-well for protein, qRT-PCR and RNA-sequencing experiments. For electrophysiology, infected iPSCs were plated on poly-D-Lysine and Matrigel-coated 12 mm coverslips (Neuvitro, #GG-12-PDL) at 60,000 cells/coverslip in a 24-well plate. For immunofluorescence studies, infected iPSCs were plated at 5000 cells/well of a Matrigel-coated 8-Well high μ-Slide (Ibidi, #80806). For Sholl analysis, infected iPSCs were plated first in Matrigel-coated 6-well plates, induced in NIM with Doxycyline for 4 days. On day 5, cells were plated into Matrigel-coated 8-Well high μ-Slide with 5000 cells/well and differentiated in NDM.

NPC-iNeurons: The numbers of NPC-iNeurons plated for RNA-sequencing, western blot, electrophysiology and immunofluorescence are the same as NGN2-iNeurons. For Sholl analysis, NPCs were plated at 5000 neurons/well of Matrigel-coated 8-Well high μ-Slide.

RNA sequencing analyses

For G118E, control, and G118E-E2KO datasets, log_2-transformed gene expression values were used to calculate Pearson correlation coefficients between all samples. Genotype-level

similarities were accessed by grouping samples by genotype and calculating the average correlation between and within groups. For within-genotype similarity, self-correlations were excluded and the mean of all pairwise correlations between different samples of the same genotype was calculated. DEG analysis was performed with DESeq2, DEGs called from DESeq2-normalized values. PCA showed that in the NGN2-derived G118E, control, and G118E-E2KO iNeurons, genotype explained majority of the variance, but neuronal health (defined by experimenter based on cell survival in culture and efficiency of NGN2 viral infection based on GFP fluorescence) was also a strong factor that explained a large percent of the variance on PCA. Therefore, to collate results across clones, neuronal health was regressed out of the differential gene analysis using the following DESeq design: design = ~ GenotypeTime + RelativeNeuronHealth. This regression approach does not alter the expression values themselves. Downstream analyses were performed on the resulting datasets. For both G118E and T158M datasets, three contrasts were generated: RTT vs Control, RTT vs RTT-E2KO and RTT-E2KO vs Control. Disease gene signature was defined as the DEGs (FDR<=0.01) between RTT vs control neurons. Dot plots were generated with $\log_{10}$ normalized average expression. Percent of genes rescued was calculated based on number of DEGs in the RTT-E2KO neurons relative to RTT that changed in the same direction as in control neurons, regardless of degree of change. Magnitude of rescue was calculated using the formula: abs (expression in RTT- expression in RTT-E2KO)/abs (expression in RTT – expression in control) and rescued genes were binned into 4 quartiles ranging from 0-100% with first quartile (0-25%) being close to expression in RTT neurons and the last quartile (75-100%) being close to expression in control neurons. Gene ontology analyses were performed using HOMER

(http://homer.ucsd.edu/homer/microarray/go.html). The top significant (p-adjusted < 0.05) biological process terms were extracted per query and the $-\log_{10}$ p-adjusted values plotted.

Electrophysiology studies

For NGN2-iNs, we recorded from the set of clones that highly expressed mature neuronal markers and were electrically active at 8-9 weeks. NPC-iNs (two clones per genotype) were recorded at 16-17 weeks after confirming the expression of neuronal maturity markers. Neurons were plated on coverslips as described above and four weeks prior to recording, NDM was replaced with 50:50 mix of NDM:serum-free human astrocyte conditioned media (ACM-sf) (Sciencell, #1811-sf) and this was continued throughout the recording period. Electrophysiological properties of iNeurons plated on coverslips were recorded as follows. To minimize the potential influence of sudden osmolarity change between culture medium and the artificial cerebrospinal fluid (ACSF: 119 mM NaCl, 2.5 mM KCl, 2 mM $CaCl_2$, 11 mM glucose and 1 mM NaH_2PO_4, 26.2 mM $NaHCO_3$, 0.2 mM ascorbic acid and 1.3 mM $MgCl_2$ (pH 7.4)), cell culture medium was gradually substituted with ACSF, which was saturated with 95% O_2 and 5% CO_2. 100 μL of media was replaced with a same volume of saturated ACSF for 5 min and this procedure was repeated 5 times at RT. Cover slips were then transferred into the recording chamber immediately with continuous perfusion of saturated ACSF at RT. Recordings were performed using a patch-clamp amplifier (MultiClamp 700B Amplifier, Molecular Devices, Union City, CA) under infrared differential interference contrast optics. The intrapipette solution contained 130mM K-gluconate, 0.1 mM EGTA, 1 mM $MgCl_2$, 2 mM MgATP, 0.3 mM NaGTP, 10 mM HEPES, 5 mM NaCl, 11 mM KCl and 5 mM Na_2-phosphocreatine (pH 7.4). Microelectrodes were made from borosilicate glass capillaries and had 5-7 MΩ resistance. To

ensure we recorded from mature, healthy neurons, recordings were collected from neurons situated in a network, rather than from lone neurons. Further, cell membrane quality was checked upon patching and only neurons with a sturdy cell membrane were used for recordings, and neurons with sticky and soft membranes were discarded. The presence of charge (recorded as described below) was also used to identify mature, electrically active neurons from which we collected electrophysiology measurements. Spontaneous synaptic activity of the iNeurons was recorded in voltage-clamp mode at −70 mV. We did not include tetrodotoxin (TTX) in the recording solution to preserve ongoing network activity. As a result, the recordings contained a substantial number of polysynaptic events, which precluded reliable quantification of standard parameters such as individual event frequency and amplitude. Instead, spontaneous synaptic activity was quantified by measuring synaptic charge transfer (charge), calculated as the time integral of the recorded synaptic currents. This approach captures overall synaptic drive under conditions of intact network activity. Action potential firing was assessed in current-clamp mode, in which input–output (current–spike) relationships were generated using incremental depolarizing currents pulses (10 pA/500 ms) with the iNeurons current-clamped at -60 mV. Membrane capacitance, membrane resistance and resting membrane potential were collected in the recording process. Data were acquired with a digitizer (DigiData 1440A, Molecular Devices) and Clampfit (Version 11.2.2.17, Molecular Devices) was used for data analysis. Data were discarded when the change in series resistance was >20% during the experiment.

Neuronal Morphology analyses

NGN2-iNs were infected with AAV9-TdTomato (stock concentration 1.96×10^8 gc/mL) at MOI 10 and incubated for 3 days for sparse infection. One week post-infection, neurons were fixed in 4% PFA and stained with 300 nM DAPI (Invitrogen, D1306) by incubating for 5 minutes

followed by 3x 10-minute washes with 1x TBS. NPC-iNs were plated sparsely in 8-Well high μ-Slide (Ibidi, #80806) and at 15-16 weeks, they were fixed in 4% PFA and stained with MAP2 and DAPI (Table S4).

SUPPLEMENTARY FIGURES

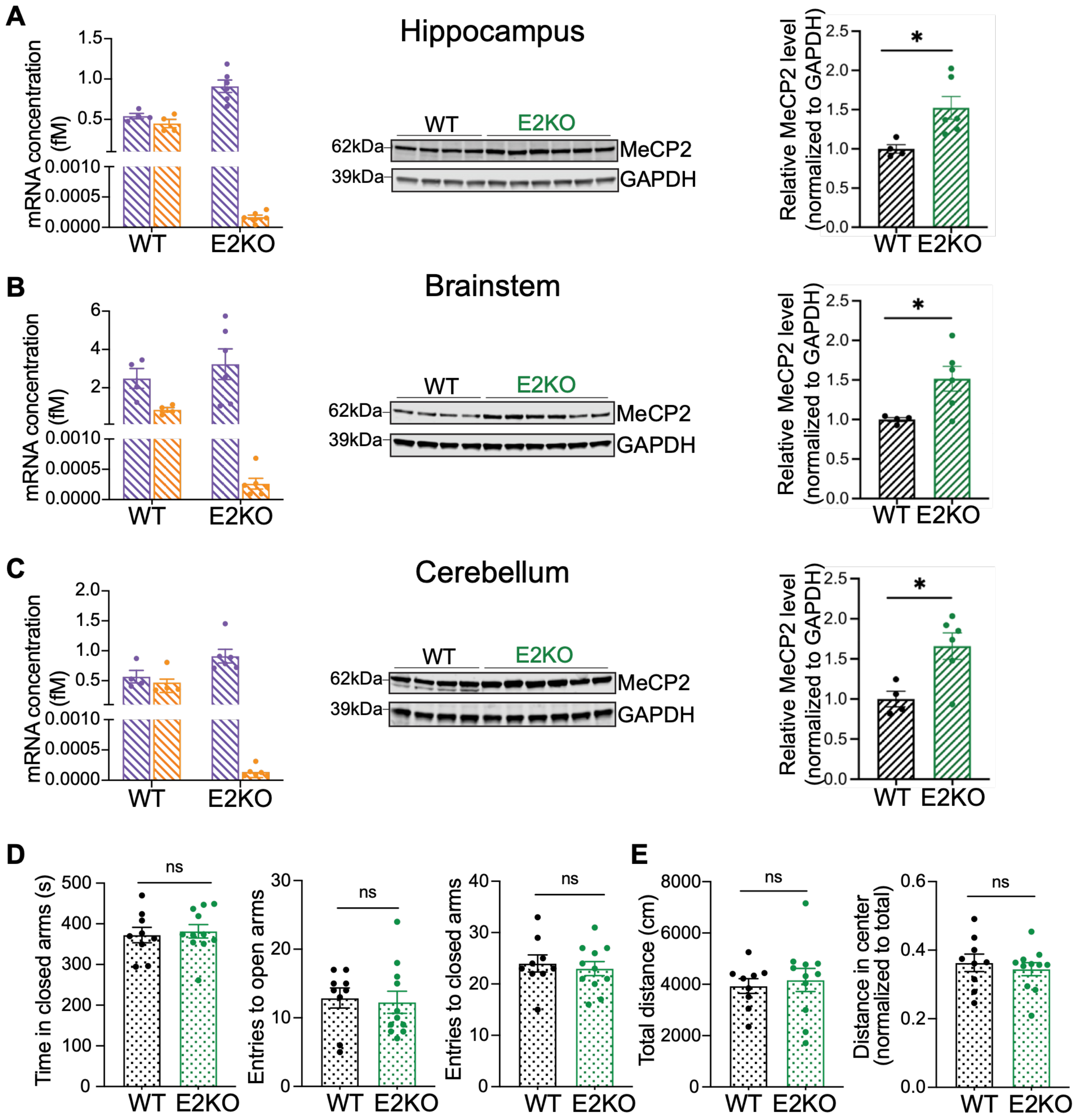

Fig. S1. Isoform switching in male mice upregulates MeCP2 protein by 50-60% and female *Mecp2*$^{E2KO/+}$ mice do not display behavioral deficits. (A-C) Left- Absolute concentration of *Mecp2-e1* and *e2* mRNA in wild-type (n=4) and E2KO (n=6) male mice (A) hippocampi, (B) brainstems and (C) cerebella; center – Western blot bands showing MeCP2 protein in wild-type and E2KO mice (A) hippocampi, (B) brainstems and (C) cerebella, right – quantification of MeCP2 protein in wild-type (n=4) and E2KO (n=6) mice (A) hippocampi, (B) brainstems and (C) cerebella, (D) Elevated plus maze (EPM) analysis showing time in closed arms (left), number of entries into open arms (center) and closed arms (right) (E) Open field assay (OFA) showing total distance travelled (left) and normalized distance (distance in center/total distance) (right) in *Mecp2*$^{E2KO/+}$ female mice (n=11, green) and wild-type littermate controls (n=9, black) at 15-16 weeks of age. Statistical analyses (where significance is indicated) were performed by unpaired t-tests (ns: $p>0.05$, * $p<0.05$). Data are presented as mean±sem with error bars representing sem and individual datapoints representing biological replicates.

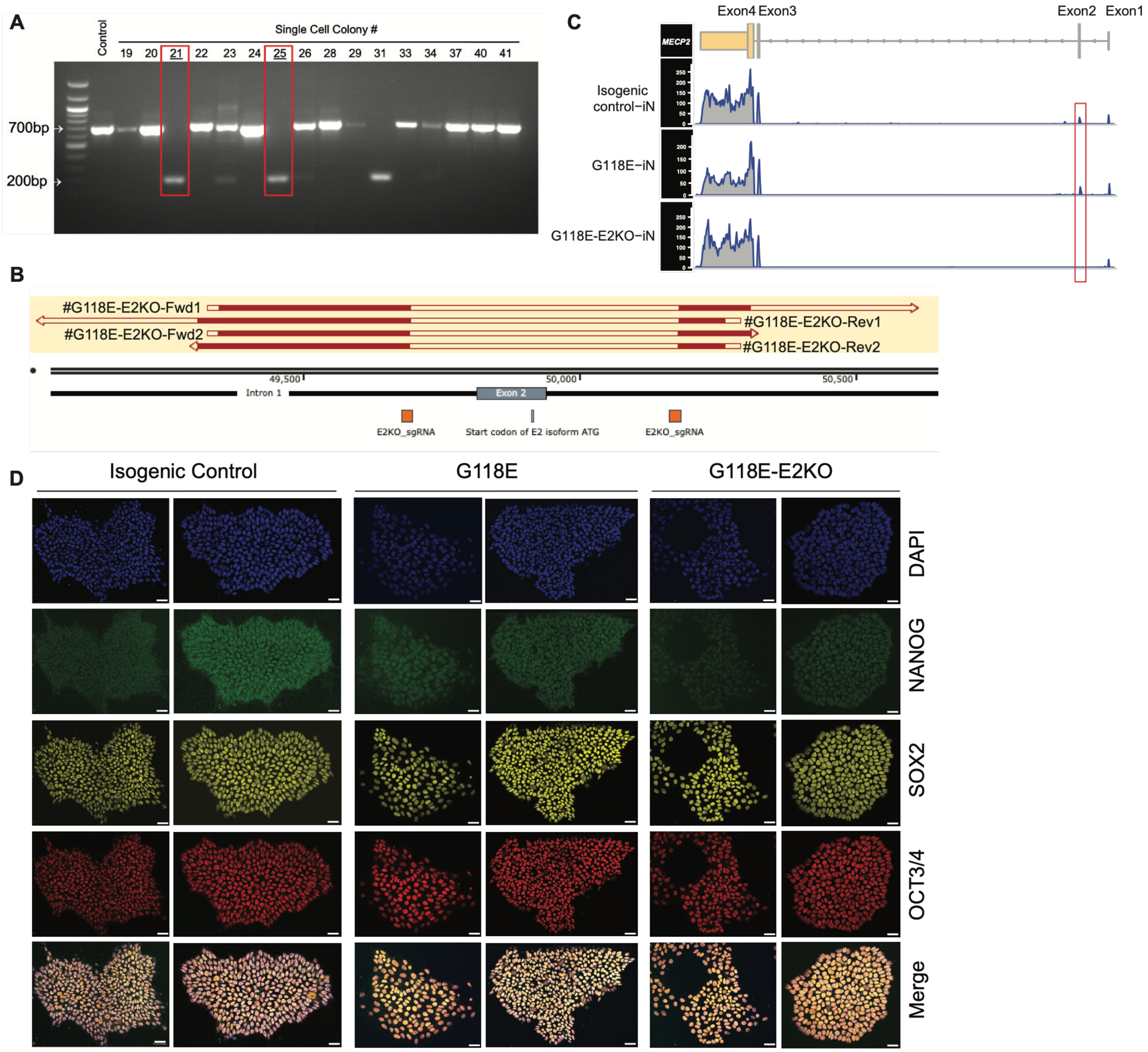
A
Control
Single Cell Colony #
19 20 21 22 23 24 25 26 28 29 31 33 34 37 40 41
700bp
200bp
B
#G118E-E2KO-Fwd1
#G118E-E2KO-Rev1
#G118E-E2KO-Fwd2
#G118E-E2KO-Rev2
49,500
50,000
50,500
Intron 1
Exon 2
E2KO_sgRNA
Start codon of E2 isoform ATG
E2KO_sgRNA
C
Exon4 Exon3
Exon2 Exon1
MECP2
Isogenic control−iN
G118E−iN
G118E-E2KO−iN
D
Isogenic Control
G118E
G118E-E2KO
DAPI
NANOG
SOX2
OCT3/4
Merge

Fig S2. G118E-E2KO iPSCs show loss of exon 2 and display pluripotency markers. (A) Agarose gel image showing bands corresponding to exon 2 PCR product in single-cell iPSC clones edited for exon 2 deletion; ~200bp band indicates exon 2 deletion and ~700bp band indicates presence of exon 2. Clones #21 and #25 (red boxes) were selected for further studies. (B) Snapshot of alignment at *MECP2* locus spanning exon 2 with sequence tracks from G118E-E2KO iPSCs (labelled 1 and 2 with one track for forward and one track for reverse primer). Filled-in maroon indicates aligned regions and clear maroon indicates gaps in sequence. Image shows gaps in exon 2 and a few basepairs flanking exon 2 in the G118E-E2KO iPSC tracks indicating clean deletion of exon 2. (C) RNA-sequencing track with the four exons of *MECP2* indicated on top for the NGN2-iNs (1 clone each of isogenic control, G118E and G118E-E2KO) showing a clear deletion of exon 2 in the G118E-E2KO iNeurons as indicated by absence of exon 2 peak (red box). (D) Immunofluorescence images of isogenic control, G118E and G118E-E2KO iPSCs showing expression of self-renewal and pluripotency markers NANOG, SOX2, OCT3/4 and nuclear stain (DAPI). Scale bar = 50 μm

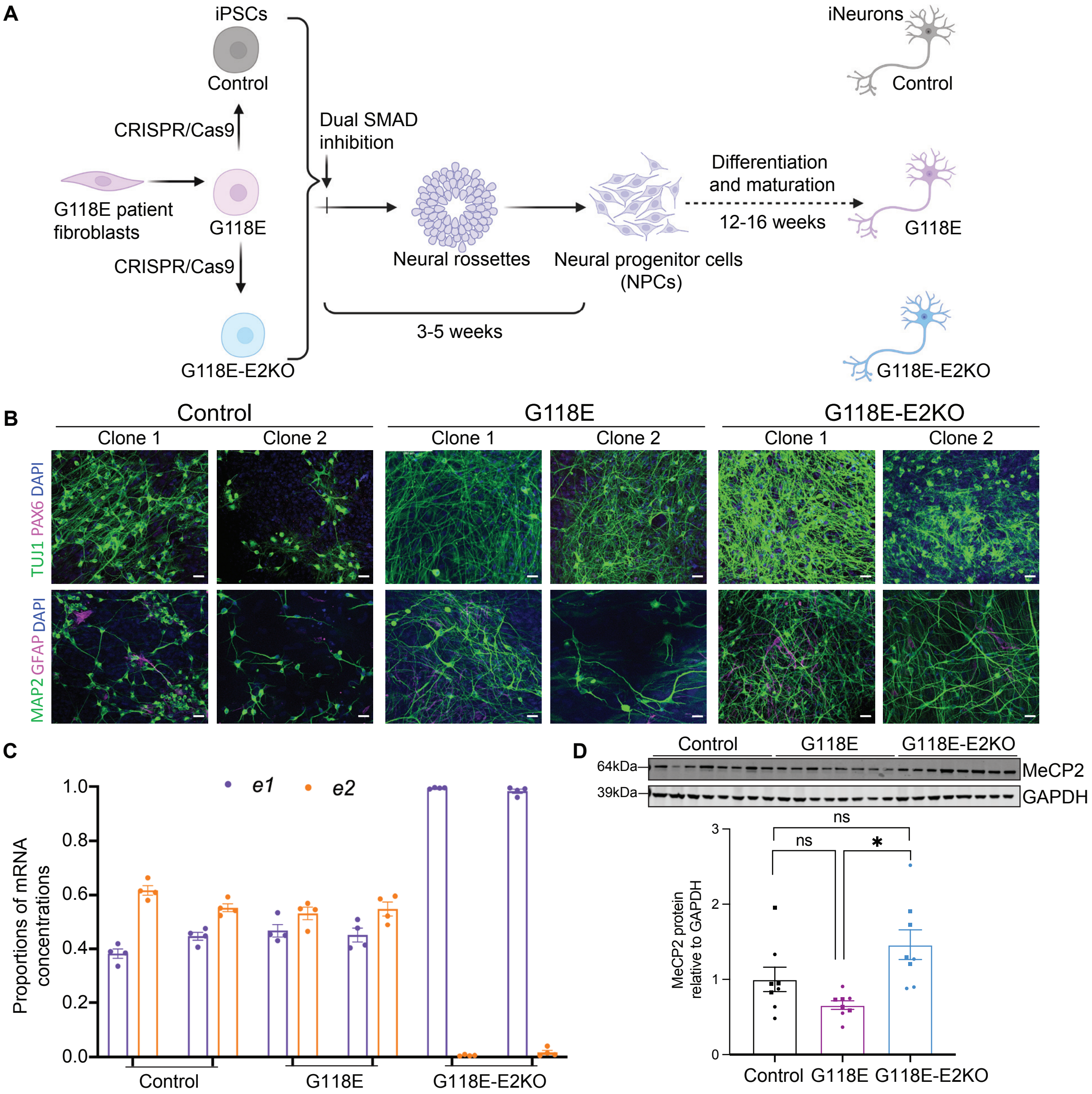
A
iPSCs
Control
CRISPR/Cas9
G118E patient fibroblasts
G118E
CRISPR/Cas9
G118E-E2KO
Dual SMAD inhibition
Neural rossettes
Neural progenitor cells (NPCs)
3-5 weeks
Differentiation and maturation
12-16 weeks
iNeurons
Control
G118E
G118E-E2KO
B
Control
G118E
G118E-E2KO
Clone 1
Clone 2
TUJ1 PAX6 DAPI
MAP2 GFAP DAPI
C
e1
e2
Proportions of mRNA concentrations
1.0
0.8
0.6
0.4
0.2
0.0
Control
G118E
G118E-E2KO
D
Control
G118E
G118E-E2KO
64kDa
39kDa
MeCP2
GAPDH
ns
ns
*
MeCP2 protein relative to GAPDH
3
2
1
0
Control
G118E
G118E-E2KO

Fig. S3. Isoform switching in G118E NPC-iNeurons upregulates MeCP2 protein. (A) Schematic of generation of G118E, control and G118E-E2KO iNeurons from RTT (G118E) patient fibroblasts by dual SMAD inhibition protocol (created with Biorender.com) (B) Immunofluorescence staining of the NPC-derived iNeurons for neuronal maturation markers at 12 weeks of differentiation in culture with the markers shown on the left in the colors corresponding to the color in images (scale bar: 50μm) (C) Proportion of *MECP2-e1* and *e2* isoforms (calculated from absolute mRNA concentrations) in the control, G118E and G118E-E2KO NPC-iNeurons with *e1* shown in purple and *e2* shown in orange as determined by qRT-PCR (D) Top – Western blot showing MeCP2 protein in control, G118E and G118E-E2KO NPC-iNeurons (n=4 per clone, n=2 clones per genotype) with GAPDH as internal control, bottom - quantification of MeCP2 protein relative to GAPDH in these iNeurons (n=4 per clone, n=2 clones per genotype). Statistical analysis in panel D was performed by nested one-way ANOVA with multiple comparisons (ns $p>0.05$, ** $p<0.01$). Data are presented as mean±sem with error bars representing sem and individual datapoints representing replicate wells (in panel D, circles denote Clone#1 and squares denote Clone#2 for each genotype).

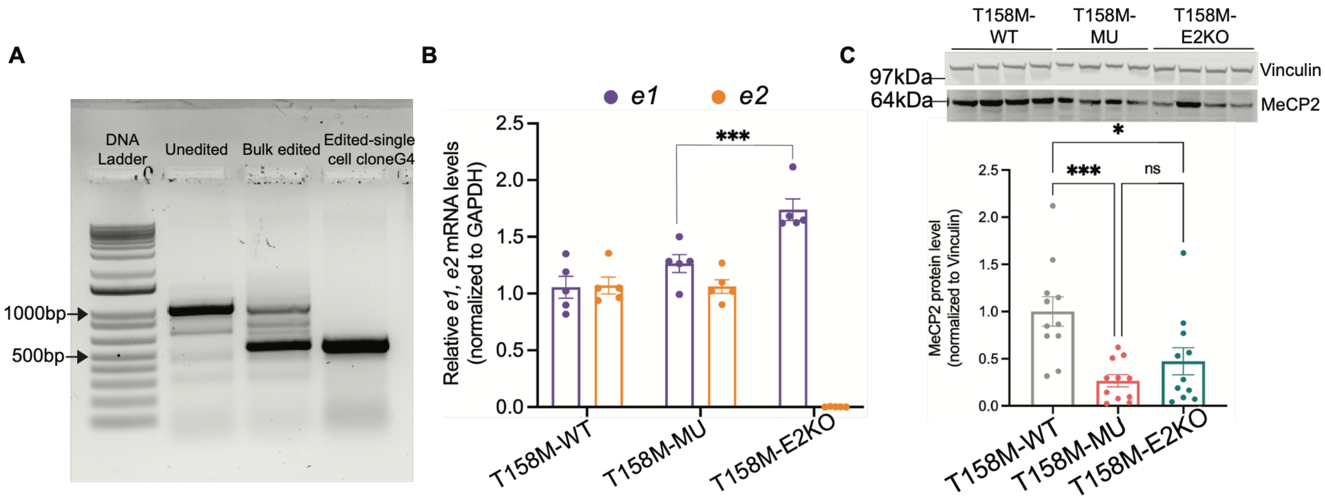
A
DNA Ladder
Unedited
Bulk edited
Edited-single cell cloneG4
1000bp
500bp
B
e1
e2
Relative e1, e2 mRNA levels (normalized to GAPDH)
2.5
2.0
1.5
1.0
0.5
0.0
***
T158M-WT
T158M-MU
T158M-E2KO
C
T158M-WT
T158M-MU
T158M-E2KO
97kDa
64kDa
Vinculin
MeCP2
MeCP2 protein level (normalized to Vinculin)
2.5
2.0
1.5
1.0
0.5
0.0
*
***
ns
T158M-WT
T158M-MU
T158M-E2KO

Fig. S4. Exon 2 deletion in T158M-NGN2-iNeurons switches *e2* to *e1* and mildly improves MeCP2 expression. (A) Agarose gel image showing bands corresponding to the PCR product of exon 2 with unedited iPSCs showing a large band (~1000bp) indicating presence of exon 2, bulk edited iPSCs showing a strong smaller band (~500bp) and a faint larger band (~1000bp) indicating a mixture of cells with and without exon 2 deletion and the single-cell iPSC clone G4 showing a clean small band indicating exon 2 deletion. The G4 iPSC line was used as T158M-E2KO for further studies. (B) Quantification of *MECP2-e1* and *e2* isoforms in the T158M-WT, T158M-MU and T158M-E2KO NGN2-iNeurons with *e1* shown in purple and *e2* shown in orange as determined by qRT-PCR (n=5 replicates per genotype, n=1 clone per genotype) (C) Top – representative Western blot showing MeCP2 in T158M-WT, T158M-MU and T158M-E2KO iNeurons with Vinculin as internal control, bottom - quantification of MeCP2 protein relative to Vinculin in the iNeurons (n=11 replicates per genotype, n=1 clone per genotype). Statistical analysis for panel B was performed with two-way ANOVA with Tukey's multiple comparisons and for panel C, ordinary one-way ANOVA with multiple comparisons was used (ns: $p>0.05$, * $p<0.05$, *** $p\leq0.001$). Data are presented as mean±sem with error bars representing sem and individual datapoints representing replicate wells.

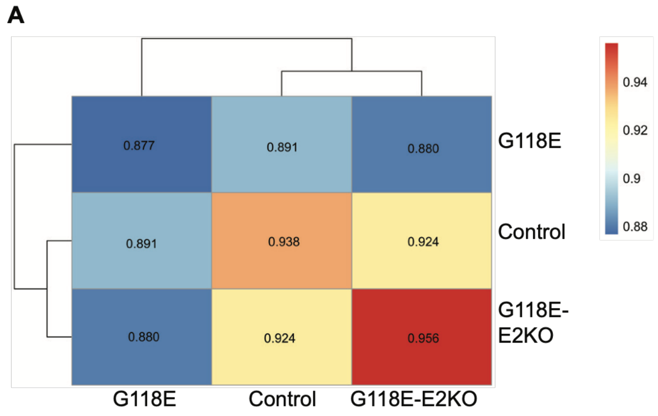
A
0.877
0.891
0.880
G118E
0.891
0.938
0.924
Control
0.880
0.924
0.956
G118E-
E2KO
G118E
Control
G118E-E2KO
0.94
0.92
0.9
0.88
B
0.849
0.864
0.862
G118E
0.864
0.907
0.904
Control
0.862
0.904
0.934
G118E-
E2KO
G118E
Control
G118E-E2KO
0.92
0.9
0.88
0.86

Fig. S5. Transcriptome of G118E-E2KO neurons is more similar to Control neurons than to G118E neurons. (A-B) Similarity matrices showing Pearson correlation coefficients between G118E, Control and G118E-E2KO NGN2-iNeurons at 4 weeks (A) and 8 weeks (B) of differentiation. Correlation coefficients were calculated with log_2-transformed normalized gene expression values of all the genes. For within-genotype similarity, self-correlations were excluded and mean of all pairwise correlations between different samples of the same genotype were calculated.

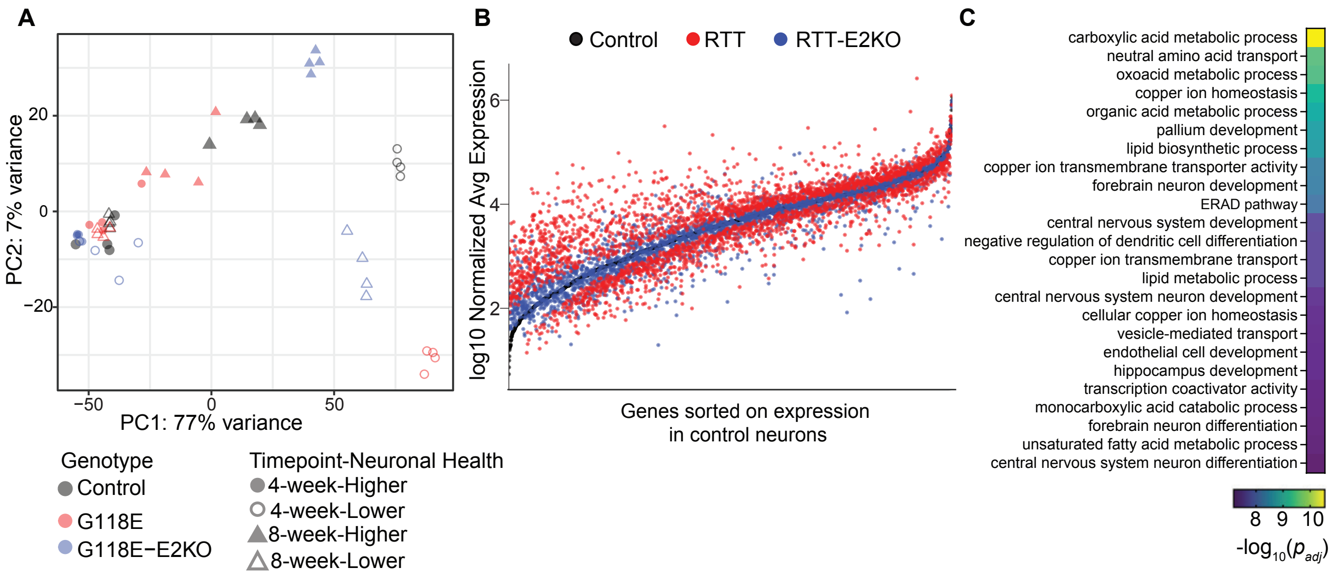

A
PC2: 7% variance
20
0
−20
−50
0
50
PC1: 77% variance
Genotype
Control
G118E
G118E−E2KO
Timepoint-Neuronal Health
4-week-Higher
4-week-Lower
8-week-Higher
8-week-Lower
B
Control
RTT
RTT-E2KO
log10 Normalized Avg Expression
6
4
2
Genes sorted on expression
in control neurons
C
carboxylic acid metabolic process
neutral amino acid transport
oxoacid metabolic process
copper ion homeostasis
organic acid metabolic process
pallium development
lipid biosynthetic process
copper ion transmembrane transporter activity
forebrain neuron development
ERAD pathway
central nervous system development
negative regulation of dendritic cell differentiation
copper ion transmembrane transport
lipid metabolic process
central nervous system neuron development
cellular copper ion homeostasis
vesicle-mediated transport
endothelial cell development
hippocampus development
transcription coactivator activity
monocarboxylic acid catabolic process
forebrain neuron differentiation
unsaturated fatty acid metabolic process
central nervous system neuron differentiation
8
9
10
-log10(padj)

Fig S6. Isoform switching corrects transcriptomic dysregulation in 4-weeks-old, G118E NGN2-iNeurons. (A) Principal component analysis plot visualizing global gene expression patterns of RNA-sequencing samples from 4-week-old (circles) and 8-week-old (triangles), G118E (red), G118E-E2KO (blue) and isogenic control (grey) NGN2-iNs designated as "lower" neuronal health (hollow dots) and "higher" neuronal health (filled-in dots) with each dot representing a replicate and their positions determined by the first two principal components (PC1 and PC2) which capture the most significant variance in the data (B) Dot plot of the G118E disease gene signature (2900 genes in total) expression with the Log_{10}Normalized average gene expression on Y-axis and the individual genes arranged in ascending order based on expression in 4-week-old control iNeurons on the X-axis. Each individual X position corresponds to one gene, with disease gene expression in control shown in black, G118E shown in red and G118E-E2KO shown in blue (C) Gene Ontology analysis of genes dysregulated in G118E and corrected in the G118E-E2KO iNs at 4 weeks showing significantly enriched biological processes along the Y-axis and the color of the box indicating the corresponding $-\log_{10}$ (p_{adj}) value with the scale shown on the bottom.

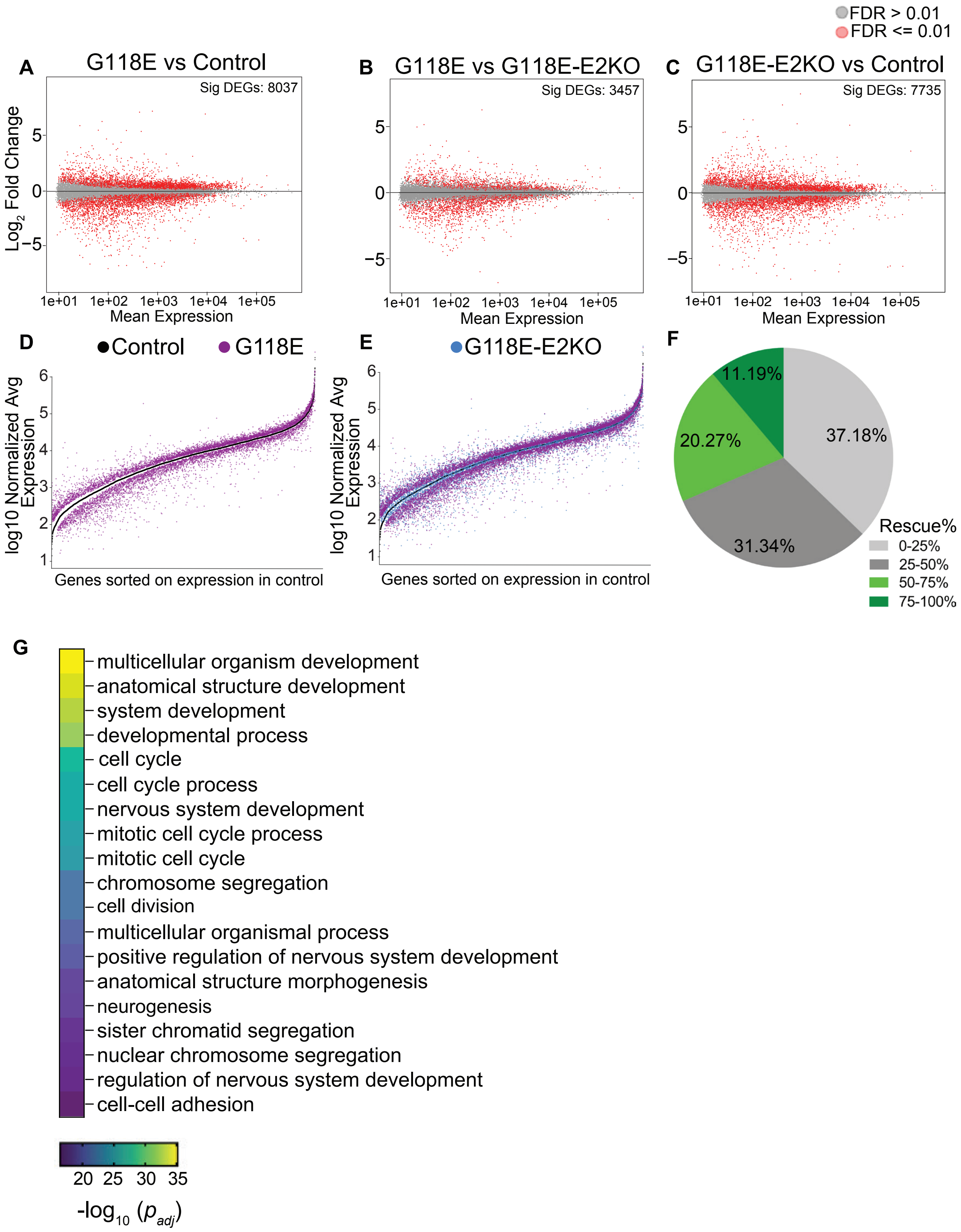
FDR > 0.01
FDR <= 0.01
A
G118E vs Control
Sig DEGs: 8037
Log_2 Fold Change
Mean Expression
B
G118E vs G118E-E2KO
Sig DEGs: 3457
Mean Expression
C
G118E-E2KO vs Control
Sig DEGs: 7735
Mean Expression
D
Control
G118E
log10 Normalized Avg Expression
Genes sorted on expression in control
E
G118E-E2KO
log10 Normalized Avg Expression
Genes sorted on expression in control
F
11.19%
37.18%
20.27%
31.34%
Rescue%
0-25%
25-50%
50-75%
75-100%
G
multicellular organism development
anatomical structure development
system development
developmental process
cell cycle
cell cycle process
nervous system development
mitotic cell cycle process
mitotic cell cycle
chromosome segregation
cell division
multicellular organismal process
positive regulation of nervous system development
anatomical structure morphogenesis
neurogenesis
sister chromatid segregation
nuclear chromosome segregation
regulation of nervous system development
cell-cell adhesion
20 25 30 35
$-\log_{10}(p_{adj})$

Fig. S7. Isoform switching corrects transcriptomic dysregulation in G118E NPC-iNeurons. (A-C) Volcano plots of DEGs between (A) G118E and control, (B) G118E and G118E-E2KO (C) G118E-E2KO and control NPC-iNs with mean expression on X-axis and Log_2 Fold change on Y-axis (D-E) Dot plots of the G118E disease gene signature (8037 genes in total) expression with Log_{10}Normalized average gene expression on Y-axis and the individual genes arranged in ascending order based on expression in control iNeurons on the X-axis. Each individual X position corresponds to one gene, with disease gene expression in control in black, G118E in purple and G118E-E2KO in blue (F) Pie-chart indicating magnitude of rescue in the corrected genes divided into four bins based on rescue percentage with each slice in a different color representing a range of rescue (light grey - 0-25%, dark grey – 25-50%, light green – 50-75% and dark green – 75-100%) and the percent of genes indicated within each bin (G) Gene Ontology analysis of genes dysregulated in G118E and corrected in the G118E-E2KO NPC-iNs showing significantly enriched biological processes along the Y-axis and the color of the box indicating the corresponding -log_{10} (p_{adj}) value with the scale shown below.

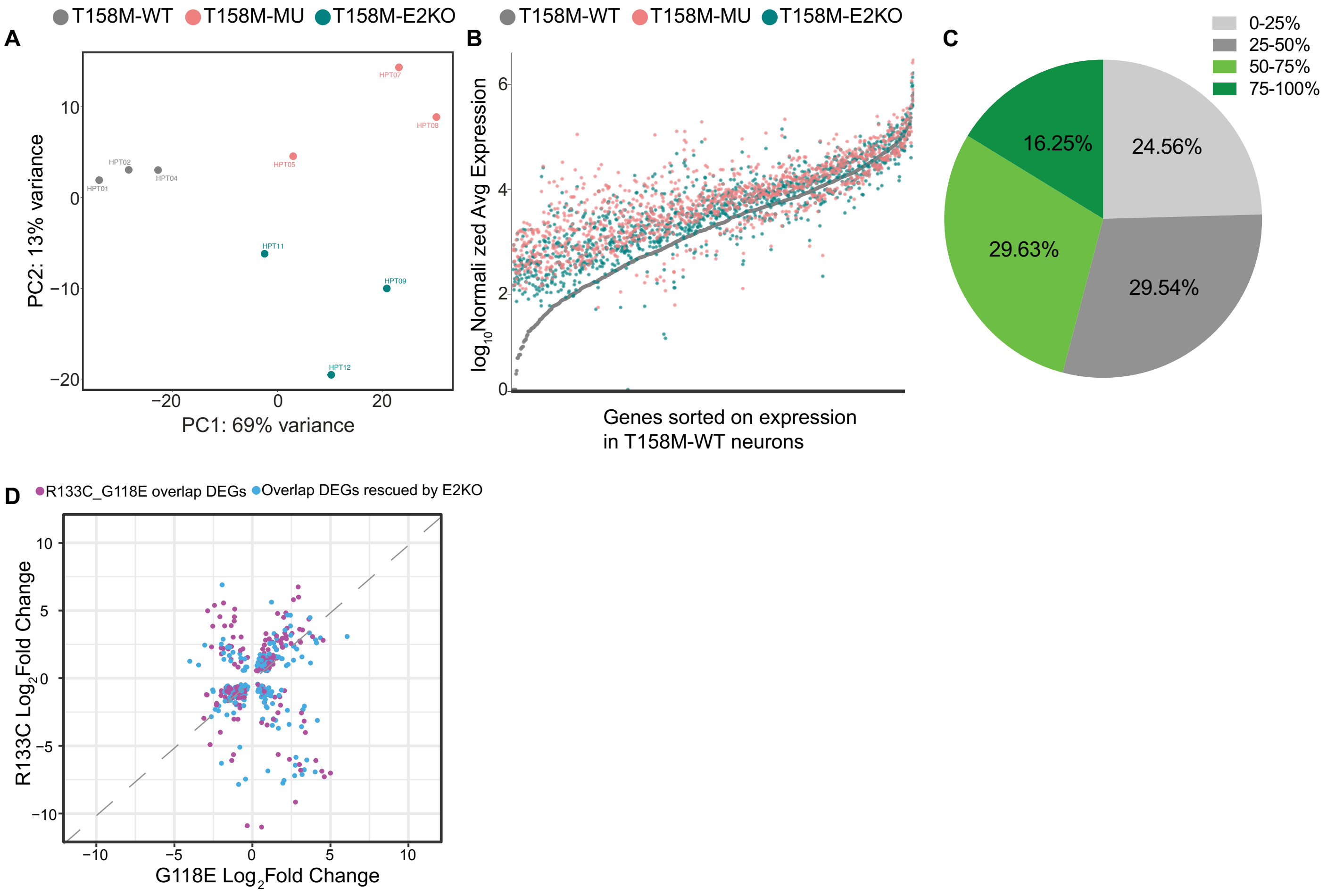

A
T158M-WT
T158M-MU
T158M-E2KO
HPT01
HPT02
HPT04
HPT05
HPT07
HPT08
HPT09
HPT11
HPT12
PC1: 69% variance
PC2: 13% variance
B
T158M-WT
T158M-MU
T158M-E2KO
$\log_{10}$Normali zed Avg Expression
Genes sorted on expression in T158M-WT neurons
C
0-25%
25-50%
50-75%
75-100%
16.25%
24.56%
29.63%
29.54%
D
R133C_G118E overlap DEGs
Overlap DEGs rescued by E2KO
R133C Log_2Fold Change
G118E Log_2Fold Change

Fig. S8. Isoform switching ameliorates transcriptomic dysregulation in T158M NGN2-iNeurons. (A) PCA plot visualizing global gene expression patterns of RNA-sequencing samples from T158M-WT (grey), T158M-MU (pink) and T158M-E2KO (teal) iNeurons; each dot represents a replicate (B) Dot plot of T158M-MU disease gene signature with Log_{10}Normalized average gene expression on Y-axis and genes arranged in ascending order of their expression in T158M-WT iNeurons on X-axis. Expression of disease gene in T158M-WT in grey, T158M-MU in pink and T158M-E2KO in teal (C) Magnitude of rescue in the corrected genes divided into four bins of a pie chart based on rescue percentage (light grey - 0-25%, dark grey – 25-50%, light green – 50-75% and dark green – 75-100%) and the percent of genes indicated within each bin. (D) Correlation analysis of Log_2 fold change for DEGs overlapping between 4-week-old G118E NGN2-iNeurons and 3-week-old R133C NGN2-iNeurons with all overlapping DEGs in pink and overlapped DEGs rescued by E2KO (from the 4-week-old G118E-E2KO iN dataset) in blue.

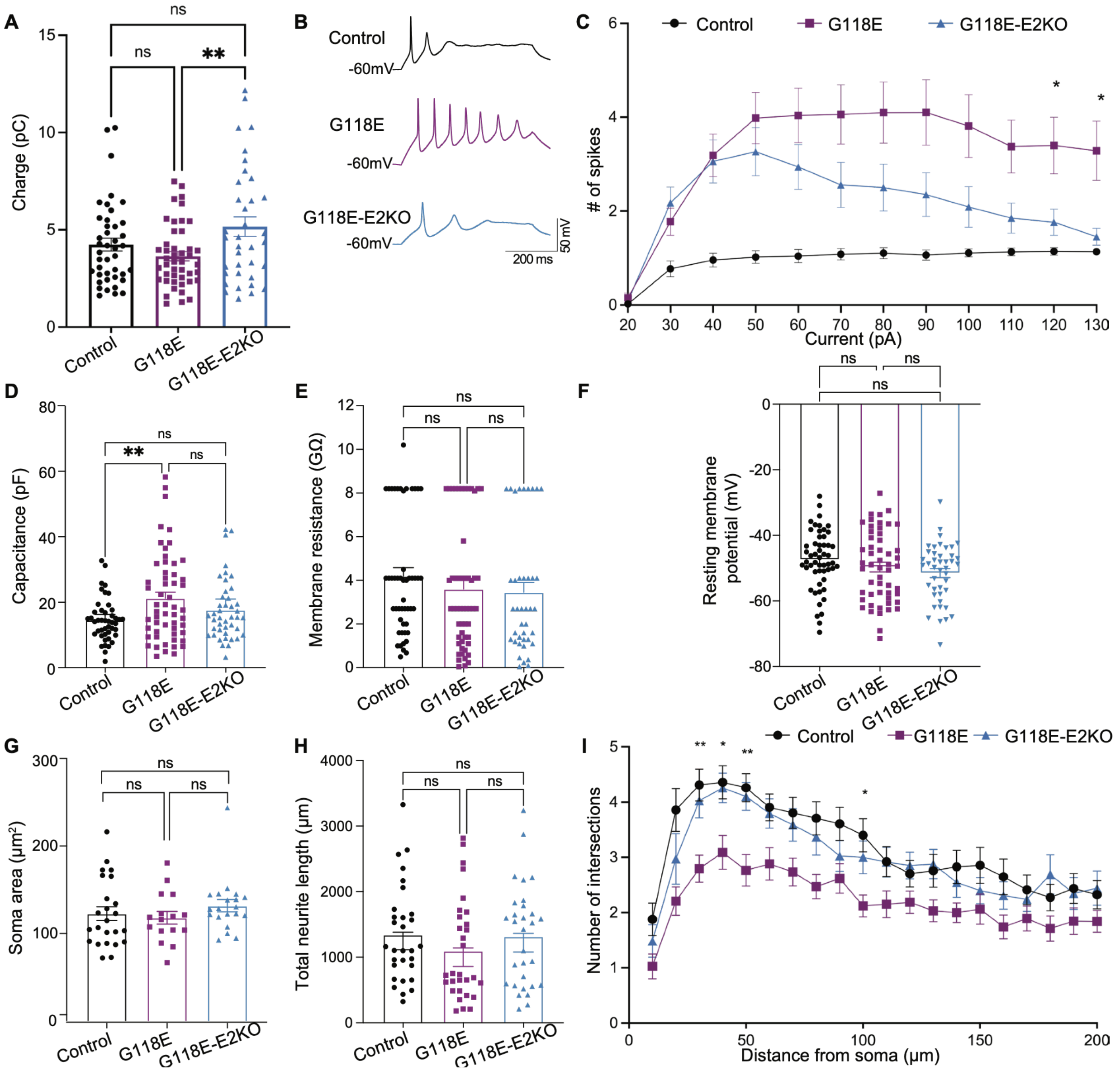

A
B
C
D
E
F
G
H
I
Charge (pC)
Control
G118E
G118E-E2KO
-60mV
200 ms
50 mV
# of spikes
Current (pA)
Capacitance (pF)
Membrane resistance (GΩ)
Resting membrane potential (mV)
Soma area (μm²)
Total neurite length (μm)
Number of intersections
Distance from soma (μm)

Fig. S9. Isoform switching ameliorates electrophysiologic and morphological deficits in G118E NPC-iNeurons. Whole-cell patch-clamp recordings of 16-17-week-old NPC-iNs to measure active and passive neuronal properties with control neurons shown in black (circles), G118E neurons in purple (squares) and G118E-E2KO NGN2-iNs in blue (triangles) (A) Charge (synaptic charge transfer, measure of spontaneous synaptic current) (B) Representative traces of current injection-dependent firing in control, G118E and G118E-E2KO iNeurons (C) Current injection-dependent firing in control, G118E and G118E-E2KO NPC-iNs in response to injection of increasing currents, (D) Capacitance, (E) Membrane resistance and (F) Resting membrane potential of control, G118E and G118E-E2KO NPC-iNs, (G) Soma area (H) total neurite length and (I) quantification of dendritic arborization with number of intersections (Y-axis) plotted at increasing distances from the soma (X-axis). Statistical analyses of cumulative action potentials (C) and dendritic arborization (I) were performed by mixed effects model with multiple comparisons (* in these panels indicates significant differences between G118E vs the other two genotypes) and for the other electrophysiology and morphology properties using ordinary one-way ANOVA with Tukey's multiple comparisons (ns: $p>0.05$, * $p<0.05$, ** $p<0.001$, ***$p<0.0001$). Electrophysiology: n=35-55 neurons recorded from 15-18 wells per genotype. Morphology: N=30-42 neurons imaged from 8-10 wells per genotype. Data are presented as mean±sem with error bars representing sem and individual datapoints representing individual neurons.

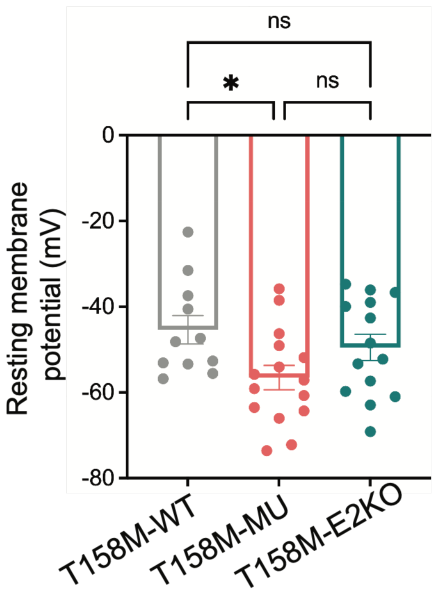

ns
*
ns
0
-20
-40
-60
-80
Resting membrane potential (mV)
T158M-WT
T158M-MU
T158M-E2KO

Fig S10. **Resting membrane potential (mV) of T158M-WT, T158M-MU and T158M-E2KO iNeurons.** Statistical analyses were performed by ordinary one-way ANOVA with Tukey's multiple comparisons (ns: $p>0.05$, * $p<0.05$) and these data are presented as mean±sem with error bars representing sem and individual datapoints representing individual neurons (n=11-15 neurons per genotype, from 4-5 wells each).

SUPPLEMENTARY TABLES

Table S1. Human postmortem tissue subject information

Case	Sex	Age (years)	Post Mort Interval (hours)	Cause of death
PDC022	M	65	12	Squamous cell carcinoma of the lung
PDC029	M	82	48	Metastatic liver and lung cancer
PDC030	M	87	17	Conductive cardiac failure; chronic kidney disease; Osteoporosis; Malignant Neoplasm of Prostate
PDC040	F	61	15	ovarian cancer
PDC026	F	80	23	Breast carcinoma with spinal metastasis; carcinosarcoma uterus

Table S2. sgRNAs and PCR primers for exon 2 deletion in human iPSCs and mice

Name	Sequence
huMECP2-E2KO-sgRNA1	5'- TTGTTTCTAGCTAGGTAAGC -3'
huMECP2-E2KO-sgRNA2	5'- TTGGTCGCCACAGCGTGATC -3'
huMECP2-E2KO-Forward Primer	5' – TCCCAGAGCACTTGGTTTCC – 3'
huMECP2-E2KO-Forward Primer	5' – AACACATGCTGAAGACCGCT – 3'
mMecp2-E2KO-sgRNA1	5'– GTCAGAAGCGTCACTATCAG – 3'
mMecp2-E2KO-sgRNA2	5' – GGTGGGTGTCAGATTAGAGC – 3'
mMecp2-E2KO-Forward Primer	5' – GGGGGAGTTGGTTGTTTTGT – 3'
mMecp2-E2KO-Reverse Primer	5' – CAGCACAGGTGAAGAGCTCA – 3'

Table S3. Primers and standard oligos for human and mouse MECP2-e1 and e2 qPCR

Name	Sequence
hMECP2_e1_Forward Primer_qRT-PCR	AGGAGAGACTGGAAGAAAAGTC
hMECP2_e1_Reverse Primer_qRT-PCR	CTTGAGGGGTTTGTCCTTGA
hMECP2_e2_Forward Primer_qRT-PCR	CCCCAGAATACACCTTGCTTCT
hMECP2_e2_Reverse Primer_qRT-PCR	CTGACTTTTCTTCCCTGAGCCC
hMECP2_e1_oligo_standard curve_qRT-PCR	CGCCGAGCGGAGGAGGAGGAGGAGGCGAGGAGGAG AGACTGGAAGAAAAGTCAGAAGACCAGGACCTCCA GGGCCTCAAGGACAAACCCCTCAAGTTTAAAAGGT GAAGAAAGATAAGAAAGAA
hMECP2_e2_oligo_standard curve_qRT-PCR	CCAGTTCCTGCTTTGATGTGACATGTGACTCCCCAGA ATACACCTTGCTTCTGTAGACCAGCTCCAACAGGATT CCATGGTAGCTGGGATGTTAGGGCTCAGGGAAGAAA AGTCAGAAGACCAGGACCTCCAGGGCCTCAAGGAC A
hMECP2_total_Forward Primer_qRT-PCR	GATCAATCCCCAGGGAAAAGC
hMECP2_total_Reverse Primer_qRT-PCR	CCTCTCCCAGTTA CCGTGAAG
hGAPDH_Forward Primer_qRT-PCR	CGACCACTTTGTCAAGCTCA
hGAPDH_Reverse Primer_qRT-PCR	TTACTCCTTGGAGGCCATGT
mMecp2_e1_Forward Primer_qRT-PCR	AGGAGAGACTGGAGGAAAAGTC
mMecp2_e1_Reverse Primer_qRT-PCR	CTTAAACTTCAGTGGCTTGTCTCTG
mMecp2_e2_Forward Primer_qRT-PCR (newFP2)	TTAGGGCTCAGGGAGGAAAAG
mMecp2_e2_Reverse Primer_qRT-PCR (newRP2)	CGCCTTCTTAAACTTCAGTGGC
mMecp2_e1_oligo_standard curve_qRT-PCR	AGCGGAGGAGGAGGAGGAGGCGAGGAGGAGAGACT GGAGGAAAAGTCAGAAGACCAGGATCTCCAGGGCC

	TCAGAGACAAGCCACTGAAGTTTAAGAAGGCGAAG AAAGACAAGAAGGAGG
mMecp2_e2_oligo_standard curve_qRT-PCR	CCATAAAAATACAGACTCACCAGTTCCTGCTTTGAT GTGACATGTGACTCCCCAGAATACACCTTGCTTCTGT AGACCAGCTCCAACAGGATTCCATGGTAGCTGGGAT GTTAGGGCTCAGGGAGGAAAAGTCAGAAGACCAGG ATCTCCAGGGCCTCAGAGACAAGCCACTGAAGTTTA AGAAGGCGAAGAAAGAC
TmMecp2_total_Forward Primer_qRT-PCR	GGCCGATCTGCTGGAAAGTA
mMecp2_total_Reverse Primer_qRT-PCR	AGGGTCCAAGGAGGTGTCTC
mGapdh_Forward Primer_qRT-PCR	GGCATTGCTCTCAATGACAA
mGapdh_Reverse Primer_qRT-PCR	CCCTGTTGCTGTAGCCGTAT

Table S4. Antibodies used for immunofluorescence studies

**Primary Antibodies [Concentration]**	**Catalog number & Company (RRID, if applicable)**
MAP2 [1:1000]	ab32454; Abcam
GFAP [1:1000]	G3893; Sigma Aldrich
PAX6 [1:500]	#100-1340, Stem Cell Technologies
TUJ1 [1:1000]	801201; Biolegend
**Secondary Antibodies [Concentration]**	**Catalog number & Company**
Goat anti-Rabbit IgG (H+L) Cross-Adsorbed Secondary Antibody Alexa Fluor® 488 [1:1000]	A-32731; Thermo Scientific; RRID: AB_2633280
Goat anti-Mouse IgG (H+L) Highly Cross-Adsorbed Secondary Antibody, Alexa Fluor 555 [1:1000]	A-21424; Thermo Scientific; RRID: AB_141780
Goat anti Guinea Pig IgG (H+L) Secondary Antibody, Alexa Fluor 647 [1:1000]	A-21450; Thermo Scientific; RRID: AB_2535867
